# Protocol: the International Milk Composition (IMiC) Consortium - a harmonized secondary analysis of human milk from four studies

**DOI:** 10.3389/fnut.2025.1548739

**Published:** 2025-06-10

**Authors:** Kelsey Fehr, Andrew Mertens, Chi-Hung Shu, Trenton Dailey-Chwalibóg, Liat Shenhav, Lindsay H. Allen, Megan R. Beggs, Lars Bode, Rishma Chooniedass, Mark D. DeBoer, Lishi Deng, Camilo Espinosa, Daniela Hampel, April Jahual, Fyezah Jehan, Mohit Jain, Patrick Kolsteren, Puja Kawle, Kim A. Lagerborg, Melissa B. Manus, Samson Mataraso, Joann M. McDermid, Ameer Muhammad, Payam Peymani, Martin Pham, Setareh Shahab-Ferdows, Yasir Shafiq, Vishak Subramoney, Daniel Sunko, Laeticia Celine Toe, Stuart E. Turvey, Lei Xue, Natalie Rodriguez, Alan Hubbard, Nima Aghaeepour, Meghan B. Azad

**Affiliations:** ^1^Department of Pediatrics and Child Health, University of Manitoba, Winnipeg, MB, Canada; ^2^Manitoba Interdisciplinary Lactation Centre (MILC), Children’s Hospital Research Institute of Manitoba, Winnipeg, MB, Canada; ^3^Division of Epidemiology, School of Public Health, University of California Berkeley, Berkeley, CA, United States; ^4^Department of Anesthesiology, Pain, and Perioperative Medicine, Stanford University School of Medicine, Stanford, CA, United States; ^5^Department of Food Technology, Safety and Health, Faculty of Bioscience Engineering, Ghent University, Ghent, Belgium; ^6^Agence de Formation de Recherche et d’Expertise en Santé Pour l’Afrique (AFRICSanté), Bobo-Dioulasso, Burkina Faso; ^7^Institute for Systems Genetics, New York Grossman School of Medicine, New York University, New York, NY, United States; ^8^Department of Microbiology, New York Grossman School of Medicine, New York University, New York, NY, United States; ^9^Department of Computer Science, New York University, New York, NY, United States; ^10^Department of Nutrition, University of California Davis, Davis, CA, United States; ^11^United States Department of Agriculture, Agricultural Research Service, Western Human Nutrition Research Center, Davis, Davis, CA, United States; ^12^Translational Medicine, The Hospital for Sick Children, Toronto, ON, Canada; ^13^Department of Nutritional Sciences, University of Toronto, Toronto, ON, Canada; ^14^Larsson-Rosenquist Foundation Mother-Milk-Infant Center of Research Excellence, University of California San Diego, La Jolla, CA, United States; ^15^Department of Pediatrics, University of California San Diego, La Jolla, CA, United States; ^16^School of Nursing, Faculty of Health and Social Development, University of British Columbia, Vancouver, BC, Canada; ^17^Department of Pediatrics, Division of Pediatric Endocrinology, University of Virginia School of Medicine, Charlottesville, VA, United States; ^18^Department of Pediatrics, Stanford University School of Medicine, Stanford, CA, United States; ^19^Department of Biomedical Data Science, Stanford University School of Medicine, Stanford, CA, United States; ^20^Department of Paediatrics and Child Health Medical College, The Aga Khan University, Karachi, Pakistan; ^21^Sapient Bioanalytics, LLC, San Diego, CA, United States; ^22^Cytel, Pune, India; ^23^Department of Anthropology, University of Texas at San Antonio, San Antonio, TX, United States; ^24^Consultant, Charlottesville, VA, United States; ^25^Vaccines and Other Initiatives to Advance Lives (VITAL) Pakistan Trust, Karachi, Pakistan; ^26^Data Aggregation, Translation and Architecture (DATA) Team, University Health Network, Toronto, ON, Canada; ^27^Department of Computer Science, University of Toronto, Toronto, ON, Canada; ^28^Center of Excellence for Trauma and Emergencies and Community Health Sciences, The Aga Khan University, Karachi, Pakistan; ^29^Harvard Humanitarian Initiative, Department of Global Health and Population, Harvard T.H. Chan School of Public Health, Cambridge, MA, United States; ^30^Global Advancement of Infants and Mothers, Department of Pediatrics, Brigham and Women’s Hospital, Boston, MA, United States; ^31^DVPL Tech, Dubai, United Arab Emirates; ^32^Unité Nutrition et Maladies Métaboliques, Institut de Recherche en Sciences de la Santé (IRSS), Bobo-Dioulasso, Burkina Faso; ^33^Department of Pediatrics, BC Children’s Hospital, University of British Columbia, Vancouver, BC, Canada

**Keywords:** human milk, breastfeeding, infant growth, infant nutrition, machine learning

## Abstract

**Introduction:**

Human milk (HM) contains a multitude of nutritive and nonnutritive bioactive compounds that support infant growth, immunity and development, yet its complex composition remains poorly understood. Integrating diverse scientific disciplines from nutrition and global health to data science, the International Milk Composition (IMiC) Consortium was established to undertake a comprehensive harmonized analysis of HM from low, middle and high-resource settings to inform novel strategies for supporting maternal-child nutrition and health.

**Methods and analysis:**

IMiC is a collaboration of HM experts, data scientists and four mother-infant health studies, each contributing a subset of participants: Canada (CHILD Cohort, *n* = 400), Tanzania (ELICIT Trial, *n* = 200), Pakistan (VITAL-LW Trial, *n* = 150), and Burkina Faso (MISAME-3 Trial, *n* = 290). Altogether IMiC includes 1,946 HM samples across time-points ranging from birth to 5 months. Using HM-validated assays, we are measuring macronutrients, minerals, B-vitamins, fat-soluble vitamins, HM oligosaccharides, selected bioactive proteins, and untargeted metabolites, proteins, and bacteria. Multi-modal machine learning methods (extreme gradient boosting with late fusion and two-layered cross-validation) will be applied to predict infant growth and identify determinants of HM variation. Feature selection and pathway enrichment analyses will identify key HM components and biological pathways, respectively. While participant data (e.g., maternal characteristics, health, household characteristics) will be harmonized across studies to the extent possible, we will also employ a meta-analytic structure approach where HM effects will be estimated separately within each study, and then meta-analyzed across studies.

**Ethics and dissemination:**

IMiC was approved by the human research ethics board at the University of Manitoba. Contributing studies were approved by their respective primary institutions and local study centers, with all participants providing informed consent. Aiming to inform maternal, newborn, and infant nutritional recommendations and interventions, results will be disseminated through Open Access platforms, and data will be available for secondary analysis.

**Clinical trial registration:**

ClinicalTrials.gov, identifier, NCT05119166.

## 1 Introduction

Human milk (HM) is the primary source of nutrition for infants and contains a plethora of non-nutritive bioactive compounds that support growth, immunity, and development—including hormones, growth factors, enzymes, antibodies, probiotic bacteria and prebiotic oligosaccharides (1, 2). The World Health Organization (WHO) recommends exclusive breastfeeding until 6 months of age, and continued breastfeeding until at least 2 years of age (3). Despite their critical role in human development and lifelong health (4), the diverse components of HM and their collective biological functions and variation remain poorly understood (5–10).

**Figure 1:**
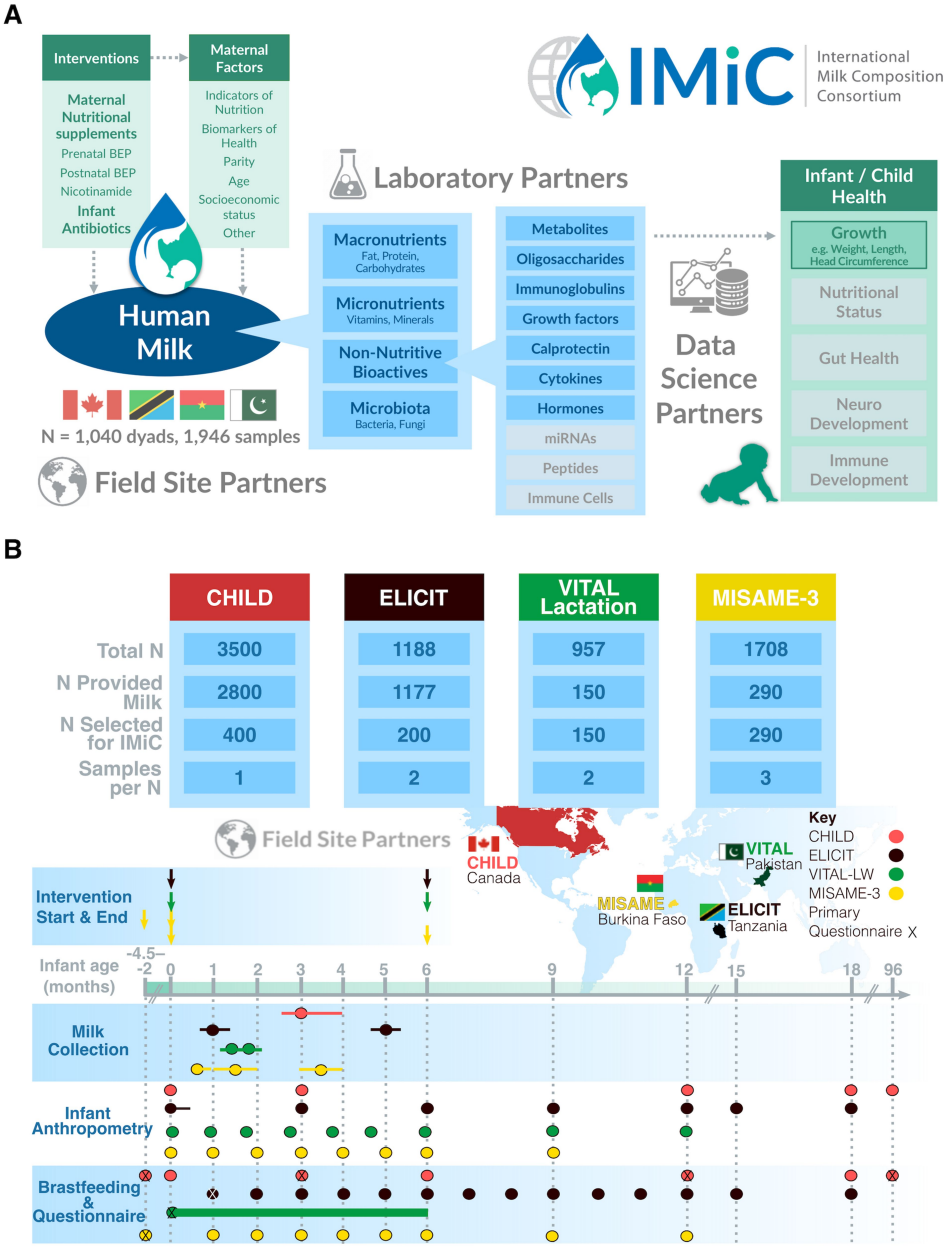
IMiC consortium research framework **(A)** and overview of participating studies **(B)**. Only the nutritional interventions provided to breastfeeding mothers are shown (ELICIT also provided a nutritional intervention to the child from 6 to 18 months). Variation in milk sample collection timing is shown as bars extending from points. CHILD had additional anthropometric measurements at 24, 30, 36, and 48 months, and questionnaires at 2, 2.5, 3, 4, and 5 years that are not shown here. Postnatal questionnaire time-points shown here included questions about breastfeeding practices. “X” indicates a primary questionnaire time-point that included additional maternal, environmental, demographic and/or socioeconomic related questions (e.g., subject-level data not expected to change over the course of the study). VITAL-LW has many follow-ups for time-varying factors (breastfeeding and child health), and therefore the questionnaire data is shown as a continuous bar. For example, breastfeeding practices are recorded on about 20–30 days between birth and 6 months.

The International Milk Composition (IMiC) Consortium was established in 2019, with the overarching goal of performing a comprehensive harmonized analysis of HM from over 1,000 mother-infant dyads living in diverse low, middle and high resource settings. Specifically, IMiC aims to characterize fixed and modifiable determinants of HM variability and identify HM components linked to infant growth (Figure 1A). IMiC is funded by the Bill & Melinda Gates Foundation and registered at ClinicalTrials.gov (NCT05119166). Below, we briefly describe the current literature and gaps in knowledge on maternal and environmental determinants of variation in HM composition and its effects on infant growth globally.

### 1.1 Macronutrients

HM provides complete nutrition in the first months of life and remains an important energy source as breastfeeding continues (11, 12). HM macronutrient profiles have been well studied, though many aspects regarding their relationship with infant growth are still unclear (9).

Apart from water, carbohydrates are the most abundant component of HM, making up about 7% of the total volume and accounting for 40%–50% of its energy content (13). Notably, only about 5% of HM carbohydrates are digestible [primarily lactose (14)]; the remainder are non-digestible HM oligosaccharides (HMOs, described below) that do not directly provide energy for the infant, but have other important bioactive functions (15). HM fat, accounting for about 3–4% of HM volume and 40–50% of its energy content (16), is primarily composed of triglycerides (98%). Triglycerides are in turn composed of fatty acids (2), which are important for cognitive and immune development (16). HM proteins account for about 1% of HM volume and 8% of its energy content (16). HM proteins can be broken down into amino acids, and along with some free amino acids in HM, utilized as building blocks for protein synthesis. Excess dietary protein may also be used as energy for growth, although this relationship has primarily been studied in formula-fed infants (17). Many HM proteins (over 1,000 identified to date) also have important non-nutritive functions in the breastfed infant (described below).

In our recent systematic review of HM macronutrients and infant growth (n = 57 studies involving 5,979 mother-infant dyads) (9), digestible carbohydrate concentrations were generally positively associated with infant weight, while protein concentrations were associated with infant length. HM fat was not consistently associated with infant growth metrics, though various associations were reported in single studies.

### 1.2 Micronutrients

Dietary micronutrients (i.e., vitamins and minerals) are essential for many biological and metabolic pathways, and thus many are associated with child growth and development (18, 19). However, based on current research, it is unclear whether and how the concentrations of micronutrients in HM influence growth during infancy. In another systematic review (*n* = 28 studies involving 2,526 dyads) we identified evidence that HM iodine, calcium and zinc concentrations may be positively associated with infant growth, but these relationships were unclear due to methodological limitations and, for many other nutrients (e.g., B vitamins, iron, sodium, potassium) data were scarce and impossible to synthesize (7).

### 1.3 Non-nutritive bioactive components

HM contains many non-digestible components that drive infant development (14). These compounds can either act locally in the infant gut or systemically at other body sites, after being absorbed across the relatively permeable infant gut barrier. HMOs are highly abundant bioactive compounds in HM, functioning as prebiotics to promote the development of commensal infant gut microbiota (15), protect against pathogens, and modulate host immunity (20). Also, many HM proteins have bioactive functions, impacting the infant immune system or directly providing protection against pathogens (21–23). For instance, lactoferrin-derived peptides have antimicrobial properties that can protect against mucosal pathogens (24). Other bioactive HM proteins that may impact infant immunity and/or metabolism include immunoglobulins, cytokines, lysozyme, growth factors and hormones (23, 25).

Additionally, HM contains derivatives of maternal fat, protein and carbohydrate metabolism (e.g., triglycerides, cholesterol, phospholipids, free fatty acids, and free amino acids), and thousands of other small molecules. Many of these metabolites have bioactive properties, and some have been associated with infant weight gain in previous research (26). Finally, HM contains a low-biomass, though relatively diverse, microbiome, that may help seed the infant gut microbiome (27–30), which plays a role in infant metabolism (31), and has potential influences on infant growth.

Our systematic review identified a seemingly vast but ultimately quite limited literature (*n* = 69 studies 9,980 dyads) exploring how a wide variety of non-nutritive HM components are related to infant growth (8). We found evidence that HM leptin, adiponectin and interleukin-6 may be associated with infant growth (8, 32), with no consistent evidence for other bioactive proteins, metabolites or HMOs.

### 1.4 HM complexity, variation and relationships with infant growth

As summarized above, limited and inconclusive evidence exists on the association of HM composition and infant growth (7–9). Additionally, many HM components are known to be highly variable, changing throughout lactation, differing across geographic settings, and varying greatly among mothers based on genetic, environmental and lifestyle factors (14). For instance, many micronutrients decrease in concentration over the course of lactation, while some are responsive to dietary interventions (1, 33–35). HMOs and microbiota appear to be affected by multiple maternal and environmental factors (e.g., geographic location, season of collection, breastfeeding exclusivity) (36–39). HM hormones differ according to maternal body composition (40, 41), and antibody levels fluctuate based on both maternal and infant health status (42). Overall, the sources of variation in HM composition and their consequences for infant growth and development remain unclear—yet this information is critical to understanding why some HM-fed infants achieve optimal growth trajectories and remain healthy, while others do not.

Another important limitation of existing HM research is that it has traditionally focused on the individual components of milk separately—an approach that does not allow investigation of the complex and dynamic HM system. For example, our systematic review identified 28 studies on HM micronutrients and infant growth (7), of which only one analyzed data from multiple micronutrients simultaneously, let alone other HM components (43). To better understand how the multitude of different HM components co-exist and interact to collectively shape infant development, a systems biology approach is required (44, 6) and careful consideration of the maternal and environmental context is essential (45, 46).

### 1.5 HM in low resource settings

Finally, apart from micronutrient studies, the vast majority of HM research has been conducted in high resource settings. For instance, less than half of studies in our systematic review of HM macronutrients (17/59, 29%) and bioactives (33/76, 43%) were conducted in low or middle-resource countries (8, 9, 32), leaving many open questions about whether and how HM composition differs in these settings, and how it relates to conditions that impact maternal, environmental, and infant health (e.g., undernutrition, poor sanitation, growth faltering). This is a critical knowledge gap from a global health perspective because breastfeeding shows a protective effect against infant mortality and infectious disease in low resource settings (4, 47), and there is a need to understand why.

### 1.6 IMiC objectives and approach

To address the above gaps, IMiC aims to develop a harmonized systems biology approach to studying HM composition in order to: (1) identify the distributions and inter-correlations of HM components across different geographic settings, including lower-middle resource settings, (2) identify maternal, nutritional and environmental sources of variation in HM composition in different geographic and resource settings, (3) evaluate how maternal nutritional interventions impact HM composition, and (4) assess how variation in HM composition is associated with infant growth in different settings (Box 1). The overarching goal of the IMiC consortium is to use the knowledge generated from answering these questions to inform maternal, and infant nutritional recommendations and interventions. A secondary objective is to evaluate whether HM components mediate the effect of nutritional interventions on infant growth outcomes.

Our Team Science approach (Figure 1A) combines HM samples and data from diverse settings (Tanzania, Burkina Faso, Pakistan and Canada) with expertise from a wide range of scientific disciplines (human milk science, nutrition, global health, epidemiology, maternal and child health, proteomics, metabolomics, immunology, microbiology, biostatistics, research operations, data management) to comprehensively analyze diverse HM components (described above) and understand their collective association with harmonized measures of infant growth. Field site partners are engaged by including principal investigators as IMiC consortium members and inviting their local colleagues to join IMiC activities.

BOX 1 **Research questions addressed in the International Milk Composition (IMiC) Consortium.**

1) How do the ranges and distributions of nutritive and non-nutritive HM components vary across different geographic settings, including lower-middle resource settings?

2) What maternal and environmental factors influence this variation in HM composition? Are these relationships consistent across different settingsh?

3) Do maternal nutritional interventions have a direct effect on HM composition?

4) How does HM composition and its variation relate to infant growth? Are these relationships consistent across different settings?

5) Does HM composition mediate the effect of exogenous factors (e.g., maternal nutrition status or interventions) on infant growth outcomes?

6) Are the above relationships consistent at different stages of lactation?

7) How do the diverse components of HM correlate with each other? Can we better answer the above questions by taking a systems biology approach to investigating comprehensive HM ‘profiles’ rather than discrete HM components?

## 2 Methods and analysis

### 2.1 Study settings, designs and HM collection

The IMiC consortium is an international collaboration of four mother-infant health studies across different settings (Table 1), each contributing a subset of mother-infant dyads from their total study populations: Canada (CHILD study, *n* = 400) (48), Tanzania (ELICIT study, *n* = 200 participating in IMiC, NCT03268902) (49), Pakistan (VITAL-LW, *n* = 150, NCT03564652) (48, 50), and Burkina Faso (MISAME-3, *n* = 290, NCT03533712) (51) (Table 2). Altogether these dyads are contributing 1,946 HM samples for IMiC analysis across various time-points in lactation, ranging from birth to 5 months (Figure 1B). Three of the four participating studies (all except CHILD) are randomized controlled trials aimed to determine the effect of maternal and child nutritional and prophylactic interventions on infant growth. Additional details on the design and objectives of the individual IMiC studies are described below and summarized in Table 2.

**Table 1:** Country-level socio-demographic and nutrition indicators for settings represented in the International Milk Composition (IMiC) Consortium.

Category	Indicator	Location			
		Canada	Tanzania	Pakistan	Burkina Faso
Diet	Typical diet/s	Western	Mainly cereal based with maize as a primary stable^a^	Variable (cereals, vegetables, fruit and yogurt are common)	Mainly cereal based with maize as a primary stable^b^
Seasonality	Month of birth, categorized	Winter (December–March) Spring (March–June) Summer (June–September) Fall (September–December)	Pre-Harvest (November–March) Post-Harvest (April–October)	Winter (December–February) Summer (March–June) Monsoon (July–September) Autumn (October–November)	Rainy (~May–October) Summer (November–April)
Child Malnutrition	Stunting, 0–59 months (%)	6.4 [1972]^c^	32.1 [2020]^d^	36.0 [2020]^d^	22.4 [2021]^d^
Maternal anemia	Hb < 110 g/L in pregnant women^e^ (%)	17.4 [2016]	48.0 [2016]	51.3 [2016]	57.5 [2016]
Obesity in women	BMI > 30 kg/m^2^ (%) [year, age range]	25.0 [2017, 18–59 yrs]^f^	8.0 [2016, 15–49 yrs]^g^	13.8 [2018, 15–49 yrs]^h^	7.3 [2021, 20–40 yrs]^i^
Poverty	Below international poverty line^e^ (%)	0.5 [2013]	49.1 [2011]	3.9 [2015]	43.7 [2014]
Infant feeding	BF within 1 h of birth^e^ (%)	No data	51.3 [2015]	19.6 [2018]	55.8 [2017]
	Infants exclusively BF to 6 months (%)	34.5 [2022]^j^	59.0 [2015]^c^	47.5 [2018]^c^	47.8 [2017]^c^
Nutritional programs	Antenatal iron supplementation (%)^c^	No data	80.9 [2016]	44.4 [2013]	92.6 [2010]
	Food fortification programs	Many fortification policies^k^	Wheat and maize flour (iron, zinc, B12, folate); Oils (vitamin A and vitamin E)	Wheat flour (iron, folate, B12, zinc); Oils (vitamin A and vitamin D)	Wheat flour (iron, folate); Vegetable oil (vitamin A), salt (iodine)

BF, Breastfeeding; Stunting defined as height for age z-score < −2. Values in [square brackets] indicate the year of reported values. ^a^Source: DeBoer et al. (49). ^b^Sources: Savy et al. (99) and Huybregts et al. (100). ^c^Sources: World Health Organization, Nutrition Landscape Information System (52). ^d^ Sources: United Nations International Children’s Emergency Fund, World Health Organization, World Bank (53) and United Nations International Children’s Emergency Fund (101). ^e^Sources: World Health Organization (102). ^f^Source: Statistics Canada (103). ^g^Source: Kikula et al. (104). ^h^Source: National Nutrition Survey (105). ^i^Source: ICF (106). ^j^Source: Public Health Agency of Canada (54). Canada’s Breastfeeding Progress Report 2022. ^k^Source: Canadian Food Inspection Agency (107).

**Table 2:** Study designs and inclusion criteria for four studies in the IMiC consortium.

Study characteristics	CHILD	ELICIT	VITAL Lactation	MISAME-3
	childstudy.ca	NCT03268902	NCT03564652	NCT03533712
Location	Canada, Mostly urban	Tanzania, Rural, Haydom	Pakistan, Urban, Karachi	Burkina Faso, Rural
Design	Prospective longitudinal birth cohort study of healthy term infants	RCT in a 2 × 2 factorial design of nicotinamide to mothers and infants, and antimicrobial prophylaxis to infants	3-arm RCT of BEP during lactation, and azithromycin prophylaxis to infants	4-arm RCT of BEP during pregnancy and/or lactation
Birth years	2009–2012	2017–2018	2018–2019	2020–2021
Postnatal follow up	8 years+	18 months	6 months	6 months (all)9 months (subset)
Total dyads	3,500	1,188	957	1708
N providing milk	2,800	1,177	150	290
Dyads and milk samples in IMiC (Target N)	400 dyads (~100 per site), 400 samples	200 dyads (50 per group), 400 samples	150 dyads (50 per group), 300 samples	290 dyads (~75 per group), 870 samples
Milk collection	Hand or pump expression, mixture of foremilk and hindmilk from multiple feedings	Hand expression, mid-feed	Hand expression, before feeding	Electric pump full breast expression
Milk sample timing (Actual N)^3^	1 sample: 3–4 months (399)	2 samples: 1 month (200) 5 months (200)	2 samples: 42 days (150) 56 days (150)	3 samples: 14–21 days (276) 1–2 months (287) 3–4 months (283)
Infant age at milk collection, (days)^*^	115.0 ± 35.2	27.9 ± 3.0 151.2 ± 3.6	41.9 ± 1.6 59.7 ± 6.6	18.4 ± 2.5 63.3 ± 6.9 120.7 ± 6.4
**Inclusion criteria**				
Maternal age	≥18 years	≥18 years	None specified	15–40 years
Maternal health	None specified	None specified	MUAC <23 cm Not allergic to peanuts, lentils, chickpeas or dairy	Not allergic to peanuts
Developmental timing at enrollment (Gestational age, weight, or age)	Gestation <30 weeks (Enrollment in pregnancy; dyad retained if infant born ≥ 35 weeks)	Infant weight ≥ 1,500 g (Enrollment at ≤14 days of infant age)	Infant weight ≥ 1,500 g (Enrollment within 168 h of birth)	Gestation ≤ 21 weeks (Enrollment during pregnancy)
Pregnancy or birth outcomes	Infant with no major congenital abnormalities or respiratory distress syndrome	No significant birth defect or neonatal illness	Infant with no known congenital anomaly or other severe illness Live birth (within 168 h)	Confirmed pregnancy
Multiple Gestation	Excluded	Excluded	Excluded	Excluded
Other	Willing to donate cord blood.	Initiated breastfeeding with intent to continue.	Planning to exclusively breastfeed until at least 6 months.	None

^*^Mean ± sd. BEP, balanced energy protein supplementation; RCT, randomized controlled trial. ^3^Samples used in analysis.

Notably, the four IMiC studies represent diverse socio-demographic settings with variation in health status, lifestyle and environmental factors (Table 1). For instance, the proportion of families below the international poverty line has been estimated at 49% in Tanzania, 44% in Burkina Faso, 3.9% in Pakistan and 0.5% in Canada (52). Infant stunting is similarly higher in the low-middle resource settings (32% in Tanzania, 22% in Burkina Faso, 36% in Pakistan) compared to Canada (6%) (52, 53). In contrast, exclusive breastfeeding rates are lower in Canada (35% at 6 months) (54), compared to the other countries represented (48% in Pakistan and Burkina Faso, and 59% in Tanzania) (52).

We will leverage the multi-study nature and high inter-study heterogeneity of IMiC to identify milk components robustly associated with maternal nutrition, environmental factors and infant growth outcomes. While data will be harmonized across studies to the extent possible, we will also employ a meta-structure approach to data analysis, where the impact of HM composition will be estimated separately within each study, and then meta-analyzed to combine estimates across studies (see Section 2.7).

#### 2.1.1 The CHILD Cohort study (Canada)

The CHILD Cohort study is an ongoing prospective general population-based longitudinal birth cohort. It was originally designed to identify interactions between genetics and environmental exposures in the development of asthma and allergy across 4 locations in Canada (Toronto, Edmonton, Vancouver, and Manitoba [Winnipeg, Morden and Winkler)] (48). Repeated questionnaires completed by participating families have captured a vast amount of longitudinal data on maternal physical and mental health, nutrition and body composition; family structure and lifestyle; indoor and outdoor environments; and infant/child health (55). Biological samples collected include maternal and cord blood; infant blood, urine and stool; and HM (56). Clinical assessments were completed at birth and 1, 3, 5, 8, and 13 years, with follow-up planned into early adulthood. The study recruited 3,624 pregnant women who gave birth to 3,542 healthy term singleton infants between 2009 and 2012. Using a subset of 400 dyads, IMiC will access HM samples collected during the CHILD 3-month home assessment, and data collected during pregnancy and the first year postpartum.

HM was collected as previously described (56). Briefly, each mother provided one sample of HM at 3–4 months postpartum in a sterile milk container provided by the CHILD study. To control for differences in the milk composition of fore- and hindmilk as well as diurnal variation, a mix of foremilk and hindmilk from multiple feeds during a 24-h period was collected. Hand expression was recommended, but pumping was also acceptable. The sample was not collected aseptically. Samples were refrigerated at home for up to 24 h before 10 mL was collected and aliquoted into 4 cryovials by study staff. Samples were stored at −80°C at the central CHILD biorepository in Hamilton, Canada until transportation to a central biorepository for IMiC.

#### 2.1.2 The ELICIT study (Tanzania)

The Early Life Interventions for Childhood Growth and Development in Tanzania (ELICIT) study is a double-blind, placebo-controlled randomized trial with a 2 × 2 factorial design conducted in Haydom, a rural area in Tanzania (NCT03268902). This study’s primary objective was to determine if infant antimicrobials and/or maternal and infant nicotinamide improve infant growth by the age of 18 months (49). At enrollment, dyads were randomly assigned to either the infant antimicrobial intervention and placebo, placebo and maternal and infant nicotinamide intervention, both interventions, or two placebos (no interventions). The antimicrobial intervention was given to the infant as a dose of azithromycin at 6, 9, 12, and 15 months, and a 3 day course of nitazoxanide at 12 and 15 months. The nicotinamide intervention was a daily dose of 250 mg nicotinamide to breastfeeding mothers from 0 to 6 months, followed by 100 mg daily to the child from 6 to 18 months. The study recruited 1,188 mother/infant dyads, enrolled before age 2 weeks between 5 September 2017 and 31 August 2018 and followed children through age 18 months. The primary outcomes of this trial have been published elsewhere (57). For a subset of 200 dyads, IMiC will access HM samples collected at 1 and 5 months and data collected through 18 months.

HM was collected as follows: milk samples were collected mid-feed. Mothers cleansed the breast around the areola with soap and water, rinsing with deionized water. Approximately 8 mL of milk was hand-expressed into a wide-mouthed sterile container. The container was then placed on ice before being transported to the laboratory, where milk was aliquoted, shielded from light, and kept at −80°C until transportation to a central biorepository for IMiC.

#### 2.1.3 The VITAL Lactating Women study (Pakistan)

The VITAL-Lactating Women (VITAL-LW) trial, also known as the MUMTA trial (MUMTA is an Urdu acronym for “nutritional support for lactating women with or without azithromycin”), is an assessor-blinded 3-armed randomized control trial conducted in Karachi, a peri-urban area in Pakistan (50) (NCT03564652). The primary objective was to identify the impact of a fortified, balanced energy protein (BEP) supplements consumed by lactating women on child growth outcomes, and to determine whether prophylactic antimicrobials provide added benefits to BEP supplementation. Lactating mothers were randomly assigned to 3 groups at enrollment: BEP intervention only, BEP with a single dose of prophylactic azithromycin to the infant at 42 days of age, or control (no interventions) (50). The BEP supplement was 2 sachets given daily from birth to 6 months postpartum. VITAL-LW recruited 957 breastfeeding mother-infant dyads between 2018 and 2020. Primary outcomes are published separately (58). For a subset of 150 dyads, IMiC will access HM samples collected at 42 and 56 days postpartum and data collected until 12 months.

HM was collected as follows: Immediately prior to breastfeeding, the breast around the areola was washed with warm water and soap and dried with a single-use cloth. At least 10 mL of milk was hand expressed directly into a sterile collection container and kept at 2°C–8°C in a box with ice packs during transport to a laboratory for aliquoting. Milk samples were mixed well prior to aliquoting into 4 sterile 1.5 mL cryovials and stored at −80°C until transportation to a central biorepository for IMiC.

#### 2.1.4 The MISAME-3 study (Burkina Faso)

The MIcronutriments pour la SAnté de la Mère et de l’Enfant 3 (MISAME-3) is a 4-arm randomized controlled trial conducted across 6 health centers in the Houndé region in rural Burkina Faso (NCT03533712). This study’s primary objective is to assess the effect of a balanced energy protein (BEP) supplement during pregnancy and/or lactation on birth outcomes and infant growth (51). At enrollment, women were randomly assigned to either prenatal intervention, postnatal intervention, both prenatal and postnatal intervention, or no intervention (control). The intervention was a BEP supplement taken daily by the mother, while both the intervention and control groups received iron/folic acid (IFA) tablets until 6 weeks postpartum. Prenatal BEP supplementation started at enrollment (<21 weeks gestation), and postnatal BEP supplementation started at delivery and continued for 6 months. The study recruited 1,708 pregnant women who gave birth to 1,628 singleton infants between 2020 and 2021. Primary outcomes of this trial have been published elsewhere (59, 60). For a subset of 290 dyads, IMiC will access HM samples collected at 14–21 days, 1–2 months and 3–4 months, and data collected during pregnancy and the first year postpartum.

HM was collected as previously described (61). Briefly, an electric breast pump (Medela, Baar, Switzerland) was employed for full expression from the breast that was not most recently used to feed the infant. The sample was then gently inverted to homogenize fore- and hindmilk. A total volume of 7.2 mL of milk was extracted from this full expression volume, and then aliquoted into 4 × 2 mL sterile cryotubes. Samples were stored in insulated bags with ice packs at home before being collected on the same day by study staff. Samples were then transferred to liquid nitrogen storage vessels and stored at −80°C in the health center before being transported to a central biorepository for IMiC.

Notably, the BEP supplement used in VITAL-LW was primarily derived from chickpeas and lentils whereas in MISAME-3 it was primarily derived from peanut paste. The exact formulations for each study differed, and are described elsewhere (58, 60, 62).

### 2.2 Eligibility criteria and selection of the IMiC subset

All study participants provided voluntary informed consent. Each study determined their eligibility criteria (summarized in Table 2) based on their primary study objectives. Further details on the consent process and eligibility criteria can be found in the protocols for each study (49–51, 55). Additional criteria used to select the “IMiC subset” from each study are described below. A notable but inevitable bias introduced through this selection process is that dyads who had stopped breastfeeding before the time of HM sample collection (or who never breastfed at all) could not be included.

#### 2.2.1 CHILD

A total of 2,800 CHILD mothers provided a HM sample, of which 400 were selected for IMiC. Selection was based on prioritizing dyads who had not already had their HM samples analyzed, and achieving representation across different infant growth trajectory categories, assigned using latent class trajectory analysis of WHO weight-for-age z-scores from birth until 5 years. Specifically, we followed the following steps: (1) infants without weight and/or length data at birth, 3 months or 12 months were excluded, (2) infants in the category with the most rapid growth were excluded due to low sample size, (3) all infants in the category with the second most rapid growth (*n* = 55) were included regardless of whether they had already had HM samples analyzed previously (*n* = 39) or not (*n* = 13) since this is an important yet relatively small group, (4) for selection of the remaining 348 milk samples, in addition to above mentioned exclusion criteria, HM samples that were already analyzed as part of previous CHILD studies (*n* = 1,200) were excluded and then (5) all remaining infants in the persistently overweight category (*n* = 63) were included and (6) an equal proportion of infants were selected from the remaining 4 categories (Stable −1 z-score, stable 0 z-score, low birth weight - stable, and stable 1 z-score; *n*~70 per group). Selection for these remaining 4 categories followed additional criteria: (1) priority was given to dyads that had infant gut microbiome data available or those expected to have gut microbiome data available in the future, (2) an approximately equal representation across CHILD study sites within each of the 4 categories, and (3) among Toronto infants, priority was given to infants with pulmonary function data (a clinical assessment only performed at the Toronto site).

#### 2.2.2 ELICIT

1,177 out of 1,188 women enrolled in the ELICIT study provided HM samples, and of these, 200 women were selected for inclusion in IMiC. After excluding women who did not provide HM samples at both study time points, we randomly selected an approximately equal number from each study group (*n* = 50 women × 4 intervention groups) from a larger subset of 400 mothers selected based on data completeness (infant blood samples collected and anthropometry measurements at all time points) and for an approximately even distribution over the recruitment year.

#### 2.2.3 VITAL-LW

Of the 957 women enrolled in the VITAL-LW trial, 150 (*n* = 50 × 3 intervention groups) were selected to provide HM samples for IMiC. Selection criteria for these 150 participants included agreement to provide all maternal and infant samples and availability to follow-up until at least the 6 month visit.

#### 2.2.4 MISAME-3

Of the 1,708 women enrolled in the MISAME-3 study, 290 provided milk samples and all were selected for inclusion in IMiC. This subset of mothers and their infants were part of the BioSpé sub-study of MISAME-3, contributing other biospecimen samples (i.e., plasma, blood, cord blood, feces and urine) at multiple time points (61). The BioSpé study was initiated following the completion of recruitment for the MISAME-III trial, when most participants were in their third trimester. To maximize the recruitment of participants in their second trimester, women were prioritized based on gestational age in descending order, ensuring an even distribution across all four study intervention groups. Ultimately, 309 women and their infants were enrolled into the BioSpé study, of which 290 provided milk samples.

### 2.3 Study participants

Participant demographics and growth outcomes are summarized in Table 3, showing high inter-study heterogeneity for many characteristics. Compared to the three low-middle resource studies, mothers in the Canadian CHILD cohort tend to be older with more education and higher BMIs, and were more likely to be primiparous. For instance, CHILD mothers had a mean age of 33 (±4) years and nearly half (48%) were primiparous, whereas MISAME mothers were nearly a decade younger (mean age 24 ± 6 years), yet only 26% were primiparous. All CHILD households and nearly all VITAL households (95%) had “improved water sources,” compared to just 59% in MISAME and 66% in ELICIT. CHILD mothers had a mean BMI of 24.2 (±4.7) kg/m^2^ with many classified as overweight or obese (32%) and very few underweight (<5%). Compared to CHILD, mothers from the low-middle resource studies had relatively lower mean BMIs, ranging from 19.8 in VITAL to 22.5 in ELICIT, and relatively higher prevalence of underweight (7% in MISAME, 10% in ELICIT, and 20% in VITAL where low mid-upper arm circumference (MUAC) was an eligibility criterion). Only 7% of CHILD newborns were small for gestational age, compared to 25% in MISAME and 38% in VITAL (birth weight and gestational age were unavailable for ELICIT where enrollment occurred up to 14 days after birth). By 3 months, 13 to 15% of infants were stunted in the low-middle resource studies, compared to just 2% in CHILD. The mean duration of exclusive breastfeeding was longer in the low-middle resource studies (range 5–6 months) compared to CHILD (3.2 ± 2.4 months).

**Table 3:** Participant characteristics of mother-infant dyads included among 4 studies comprising the International Milk Composition (IMiC) Consortium.

Variable	CHILD	ELICIT	VITAL-LW	MISAME-3	Overall
Location	Canada	Tanzania	Pakistan	Burkina Faso	-
Birth years	2009–2012	2017–2018	2018–2019	2021	-
N dyads in IMiC	399	200	150	290	1,039
Mother at baseline^a^					
Age	32.5 (4.4)	29.4 (7.1)	24.2 (4.9)	24.2 (5.6)	28.4 (6.6)
Parity	1.8 (0.9)	4.4 (2.6)	2.7 (1.9)	2.9 (1.8)	2.7 (2.0)
Gravida	2.2 (1.3)	-	3.1 (2.2)	3.1 (1.9)	2.7 (1.7)
MUAC (cm)	-	-	21.5 (1.2)	26.1 (2.7)	24.6 (3.2)
HGB (g/dL)	-	-	-	11.7 (1.4)	11.7 (1.4)
Weight (kg)	65.7 (14.0)	55.9 (10.1)	46.1 (5.2)	58.1 (9.6)	58.1 (12.6)
Height (m)	1.65 (0.07)	1.58 (0.06)	1.52 (0.06)	1.62 (0.06)	1.59 (0.07)
BMI (kg/m2)	24.2 (4.7)	22.5 (3.7)	19.8 (1.7)	22.0 (3.1)	22.4 (4.0)
BMI category					
Underweight	18 (5%)	21 (10%)	30 (20%)	21 (7%)	84 (8%)
Normal	249 (64%)	134 (67%)	120 (80%)	229 (79%)	673 (65%)
Overweight	82 (21%)	35 (18%)	0 (0%)	36 (12%)	133 (13%)
Obese	42 (11%)	10 (5%)	0 (0%)	4 (1%)	43 (4%)
Birth					
Cesarean delivery	82 (21%)	0 (0%)	24 (16%)	6 (2%)	112 (11%)
Rainy season^b^	-	107 (54%)	45 (30%)	227 (78%)	379 (36%)
Home and socioeconomicstatus					
Improved water^c^	399 (100%)	131 (66%)	143 (95%)	172 (59%)	845 (81%)
Separate cooking space	-	124 (62%)	142 (95%)	0 (0%)	266 (26%)
Improved flooring	399 (100%)	-	146 (97%)	175 (60%)	720 (69%)
Mother’s education (yrs)	16.8 (2.9)	6.5 (2.7)	7.7 (3.1)	3.2 (3.9)	10.1 (6.8)
Infant at birth					
Sex (Female)	188 (47%)	101 (50%)	84 (56%)	148 (51%)	521 (50%)
Gestational age (wks)	39.6 (1.4)	-	38.9 (1.5)	39.8 (1.5)	39.6 (1.5)
Gestational age method					
Ultrasound	399 (100%)	-	86 (57%)	290 (100%)	775 (75%)
Last menstrual period	0 (0%)	-	64 (43%)	0 (0%)	64 (6%)
Birth weight (g)	3,412 (517)	3,190 (510)^d^	2,790 (410)	3,013 (449)	3,163 (537)
Birth length (cm)	51.5 (2.7)	49.1 (2.2)^d^	48.2 (1.9)	48.5 (2.1)	49.8 (2.7)
Preterm (<37 weeks)	17 (4%)	-	16 (11%)	11 (4%)	44 (4%)
Small for gestational age	28 (7%)	-	57 (38%)	73 (25%)	158 (15%)
Infant at 3 months					
Weight (kg)	6.2 (0.9)	5.7 (0.7)	5.2 (0.8)	6.0 (0.8)	5.9 (0.9)
Length (cm)	61.9 (3.0)	58.6 (2.1)	57.9 (2.3)	59.2 (2.8)	59.8 (3.0)
Head circumference (cm)	40.6 (1.6)	39.7 (1.4)	38.4 (1.3)	39.5 (1.5)	39.7 (1.6)
WAZ	−0.06 (1.13)	−0.56 (1.00)	−1.22 (1.21)	−0.21 (1.05)	−0.40 (1.16)
LAZ	0.41 (1.28)	−0.93 (1.02)	−1.10 (1.13)	−0.66 (1.25)	−0.44 (1.34)
WLZ	−0.38 (1.20)	0.30 (1.33)	−0.38 (1.18)	0.51 (1.17)	0.06 (1.28)
HCAZ	0.28 (1.27)	−0.23 (1.11)	−1.14 (1.03)	−0.42 (1.09)	−0.24 (1.23)
MUACZ	-	-	12.28 (1.21)	13.13 (1.09)	12.88 (1.19)
Stunting^e^	8 (2%)	29 (14%)	23 (15%)	37 (13%)	97 (9%)
Wasting^f^	27 (7%)	7 (4%)	10 (7%)	5 (2%)	49 (5%)
Breastfeeding (BF)					
EBF duration (months)	3.2 (2.4)	5.1 (0.9)	5.9 (0.6)	5.8 (1.0)	4.7 (2.0)
BF status at 6 months					
Exclusive BF	79 (20%)	89 (44%)	141 (94%)	204 (70%)	513 (49%)
Partial BF	303 (76%)	111 (56%)	9 (6%)	85 (29%)	508 (49%)
No BF	13 (3%)	0 (0%)	0 (0%)	0 (0%)	13 (1%)

Values are *n* (%) or mean (standard deviation). BMI, Body mass index; HGB, blood hemoglobin; MUAC, Mid-upper arm circumference; HCAZ, head circumference for age z-scores; WAZ, weight for age z-score; LAZ, length for age z-score; WLZ, weight for length z-score; EBF, Exclusive breastfeeding; BF, Breastfeeding. ^a^Maternal baseline characteristics: parity at delivery, first measurement used for other characteristics (within the first 15 days after birth for ELICIT and VITAL-L and prenatal measures for MISAME-3 and CHILD). ^b^Rainy season refers to January until April for ELICIT and Late Summer/Monsoon for VITAL-L. ^c^Improved water definition adapted from WHO/UNICEF (73). ^d^Exact birth weight and length for ELICIT are unavailable, measurements were within the first 15 days after birth. ^e^Stunting = Infant LAZ < −2, ^f^Wasting = Infant WLZ < −2.

### 2.4 Participant data and data harmonization

All studies collected anthropometric measurements for mothers and infants, and questionnaire data capturing morbidities, infant feeding, and sociodemographics. Data collection methods across studies are described below and summarized in Table 4. Methodological differences between studies are not expected to pose major challenges given the data harmonization plan (see below) and the meta-analytic and machine learning approaches to data analysis that will be used to address inter-study heterogeneity and control for potential confounders (see Section 2.8).

**Table 4:** Maternal and infant data of interest in the International Milk Composition Consortium (IMiC) study and methods for their measurement, collection or intervention administration.

Measurement	CHILD (48)	ELICIT (49)	VITAL-LW (50)	MISAME-3 (52)
Maternal data				
Maternal Nutritional Interventions	None	Daily Nicotinamide Postpartum: Birth to 6 months vs. Placebo	Daily BEP Postpartum: Birth to 6 months vs. No BEP	Daily BEP Prepartum: Enrollment to Birth, and/or Postpartum: Birth to 6 months vs. No BEP
Measures of maternal anthropometry	By trained staff at home visits (ideally) or self-reported	By trained staff at home visits	By trained staff at home visits	By trained staff at healthcare centers
Maternal weight	Digital standing scale	Digital standing scale	Digital standing scale	Digital SECA scale
Maternal height	Stadiometer	Stadiometer	Stadiometer (SECA scale)	Infant/Child/Adult measuring board
Maternal mid-upper arm circumference	Not measured	Not measured	UNICEF measuring tape	SECA measuring tape
Infant data				
Measures of infant anthropometry	By trained staff at 3-month home visit and 1, 3 and 5 year clinic visits.	By trained field staff at home visits	By trained field staff at home visits.	By trained field staff at healthcare centers.
Length	Measuring board	Measuring board	Measuring board (SECA scale)	SECA Infantometer
Weight	Digital scale	Digital scale	Digital scale	SECA scale
Head circumference	Non–distensible tape	Non–distensible tape	UNICEF measuring tape	SECA measuring tape
Mid-upper arm circumference	Not measured	Non-distensible tape at mid-point of upper arm	UNICEF measuring tape	SECA measuring tape
Breastfeeding	Questionnaire at 0, 3, 6, and 12 months	Recall at 6 months	Biweekly questionnaires 1 to 6 months	Monthly questionnaire, 1 to 6 months

BEP; Balanced-energy protein supplement; BM, breastmilk.

#### 2.4.1 Anthropometric measurements

**Figure 2:**
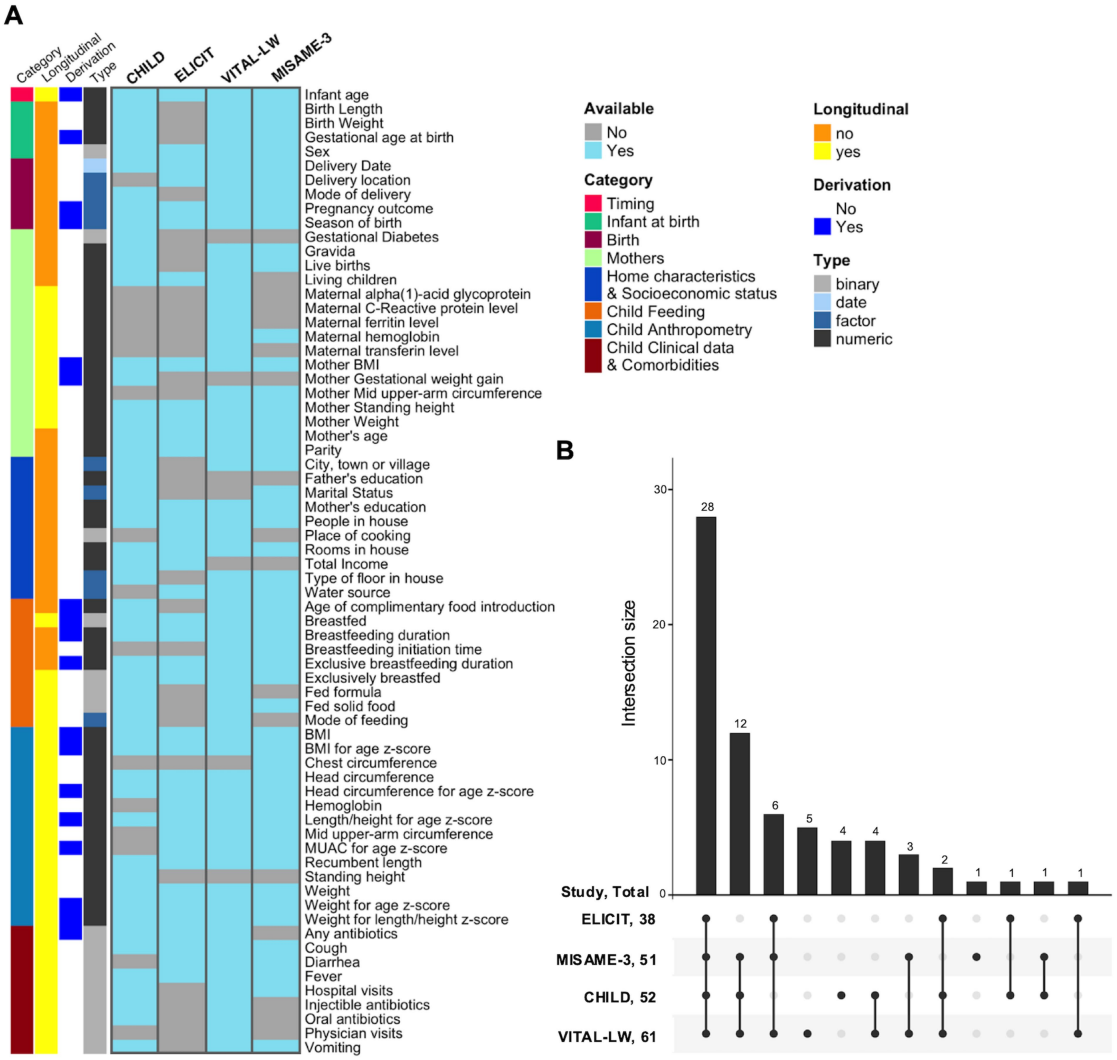
Availability of harmonized data across IMiC studies. **(A)** Note that the derivation annotation is just a general rule, there are sometimes exceptions for individual sites (see Supplementary Table S1). Only variables harmonized by the Ki Data Curation team are shown (e.g., excludes derived summary variables generated downstream for specific analyses). Other excluded variables: identifiers, method/descriptors, and analysis/derivation flags (e.g., recall, sampling, imputation flags). Also combines recall and non-recall versions of the same variable. **(B)** Number of variables available in harmonized data across IMiC studies. Excludes identifiers, method/descriptor, and analysis/derivation flags (e.g., recall, sampling, imputation flags), and combines recall and non-recall versions of the same variable.

Anthropometric measures include the infant’s length, weight, head circumference and MUAC, and the mother’s height, weight and MUAC (Figure 2A). These measurements were collected using similar methods across studies by staff members trained in measuring anthropometric indices (Table 4), either during home visits or visits of the mother and child to healthcare centers. These anthropometric measures were taken at varying intervals depending on the study, but all studies covered time points at or near birth, 3 months and 12 months of age, and all but the CHILD study also covered 6 months of age (Figure 1B). Anthropometric z-scores were derived using the WHO child growth standards (63, 64).

#### 2.4.2 Questionnaire and other clinical data

Each study provided repeated standardized questionnaires to participants that, at minimum, covered the following topics: basic demographics, socioeconomic factors, home characteristics, child feeding practices and child comorbidities (Figure 2A). Questionnaire time points varied across studies (Figure 1B), but all included time points at or near birth or enrollment, 3 and 6 months of age, with the majority following up to at least 12 months of age. Exclusive breastfeeding duration for all studies was collected until at least 6 months of age.

#### 2.4.3 Data curation, harmonization and quality control

Anthropometric, questionnaire, clinical and co-morbidity data were curated and harmonized by the Ki Data Curation team at the Bill & Melinda Gates Foundation.^1^ This process includes transforming the data from each study into a standardized format to achieve consistency across studies. The raw data for each study is first transformed into pre-defined Ki standard datasets, which are based on standards developed by the CDISC SDTM (Clinical Data Interchange Standards Consortium Study Data Tabulation Model). An IMiC Harmonized dataset is then created for each study from the standard Ki datasets. Data from all studies are then aggregated into a single IMiC harmonized dataset comprising child and maternal anthropometry, clinical data, and curated questionnaire data needed for analysis. This includes derived variables generated from multiple questions in the raw data and/or multiple data sources, to increase comparability across sites. For instance, child feeding questions were used to generate harmonized variables such as duration of exclusive breastfeeding. Supplementary Table S1 provides a complete list of harmonized variables and their availability across the IMiC studies. Data availability across studies is illustrated in Figure 2.

Clinical and questionnaire data are assessed by the Ki Data Curation team for inconsistencies and errors at various stages and final harmonized datasets go through additional quality assessments by IMiC data analysts. These assessments include but are not limited to: validating attributes, checking for duplication and impossible values, evaluating the level of data missingness, and ensuring consistency in data values before and after preprocessing. Any potential issues identified during the review processes are reported to the Ki team analysts and/or field sites to identify solutions and resolve them. Further, after data is updated, datasets are version compared to ensure only the expected changes are made.

The resulting harmonized variables will be used by analysts either directly for analysis, or to derive further variables, such as household wealth index (65), summary variables indicating improved flooring or improved water sources (66), and variables summarized across a time range (e.g., antibiotic exposure before 1 year of age). Aims of these further derivations include limiting data sparsity, reducing inter-study heterogeneity, and allowing for statistical analysis of all sites combined. Additionally, anthropometric data was further curated by the Division of Data Science and Innovation team (UC Berkeley team) to ensure data can be pooled across studies despite differences in measurement methods and time points. This includes the removal of extreme outliers (e.g., |HAZ| and |WAZ| > 6, |WHZ|, |HCAZ|, and |MUAZ| > 5) (63, 64), derivation of age-specific primary outcomes at 3, 6, 12 and 18 months, which use each participant’s measurement taken closest to the target age within a 1-month window, and the calculation of weight and height growth velocity z-scores between harmonized measurement points.

### 2.5 HM processing and distribution

HM samples were collected independently for each study as described in section 2.1, and later shipped on dry ice to a central biorepository at the Manitoba Interdisciplinary Lactation Center (MILC) in Canada. Supplementary Figure S1 shows the distribution of HM pools and sample aliquots from study sites to the MILC biorepository and then to laboratory analysis sites.

#### 2.5.1 Sample aliquoting and distribution

The MILC biorepository (Manitoba, Canada) serves as the central hub for IMiC sample storage (in alarmed −80°C freezers), aliquoting and distribution. Samples are processed and distributed with consideration for quality control (QC), freeze/thaw minimization, analysis plate layouts, and sample randomization requirements. Full aliquoting protocols are provided in Supplementary material. In brief, samples are thawed on ice and homogenized right before splitting into sterile barcoded tubes. During this process, samples are also randomized with respect to intervention groups and a few key variables (where relevant, intervention group, primiparity, study center and season of collection) based on the plate layouts used for each analysis.

#### 2.5.2 HM pools

Pooled HM samples are included as replicates on every analysis plate and/or batch for all assays, in order to determine within and between assay variation introduced by technical effects, and identify potential batch effects. Additionally, sample composition, including components other than the analyte of interest, is known to introduce a type of technical effect known as a matrix effect, which can introduce unexpected results, for instance, increased technical variation due to interference with the ionization process in MS (67, 68) Therefore, study-specific HM pools were produced where possible (i.e., for VITAL-LW and MISAME-3, the studies that were still collecting HM at the time IMiC was established). In VITAL-LW and MISAME-3, for the first 10 participants, additional milk (~10–15 mL) was collected for QC purposes. The date, time and volume of milk collected was recorded, and consistent labeling was used. In addition, a general milk pool for use across all studies and assays was created using milk from 10 mothers between 2 and 10 months postpartum (~150 mL per mother), provided by the NorthernStar Mothers Milk Bank in Canada. To create milk pools, milk was pipetted into a sterilized beaker containing a stir rod and homogenized using a stir plate (Supplementary material), then aliquoted in the same manner as study samples.

### 2.6 Laboratory analysis of HM

HM samples will be analyzed for macronutrients, micronutrients, HMOs, metabolites, proteins, and microbes by laboratories with expertise in these methods and experience applying them to HM (Table 5). This encompasses both targeted approaches for absolute quantification of specific known analytes, as well as untargeted approaches for exploratory analyses of potentially unknown and known compounds. A brief description of these analyses is provided below (Table 5).

**Table 5:** Planned analyses in the International Milk Composition Consortium (IMiC).

Analysis category and type^a^	Analyte sub-class	Analyte	Method	Ref	Laboratory
Macro-nutrients Targeted	Macronutrient	Protein	Near-infrared spectroscopy (NIR)	(69, 70)	Allen Lab, USDA, ARS-WHNRC (USA) & MILC biorepository (Canada)
		Carbohydrates			
		Fat			
Micro-Nutrients Targeted	Fat soluble vitamins	Vitamin A	HPLC multi-wavelength detection (MWL)	(75)	Allen Lab, USDA, ARS-WHNRC (USA)
		Vitamin E			
		Carotenoids			
	Water soluble vitamins	B2 (riboflavin, FAD, FMN)	Ultra performance liquid chromatography—tandem mass spectrometry (UPLC–MS/MS)	(74)	
		B3 (nicotinamide, NMN, NAD, NR, NADP)			
		B3 precursor, tryptophan			
		B3 catabolite, nudifloramide			
		B5 (pantothenic acid)			
		B6 (pyridoxal, PN, PLP)			
		B7 (Biotin)			
		B9 (Folic acid)			
		B1 (Thiamin, TMP, TDP)	HPLC-fluorescence detection (FLD)	(72)	
		B12 (Cyanocobalamin)	Competitive protein binding assay	(73)	
	Minerals	Iron	Inductively coupled plasma (ICP)-MS	(76)	
		Copper			
		Zinc			
		Selenium			
		Sodium			
		Potassium			
		Magnesium			
		Calcium			
HMOs Targeted	HMO summary analytes	Sum of all HMOs, Total	HPLC-FLD	(38, 78)	Bode Lab, UC San Diego (USA)
		HMO bound sialic acid			
		HMO bound fucose			
	Fucose bound HMOs	2′-fucosyllactose			
		3-fucosyllactose			
		Fucosyllacto-N-hexaose			
		Difucosyllacto-N-hexaose			
		Difucosyllactose			
		Difucosyllacto-N-tetrose			
		Lacto-N-fucopentaose I			
		Lacto-N-fucopentaose II			
		Lacto-N-fucopentaose III			
	Fucose & Sialic Acid bound HMO	Fucodisialyllacto-N-hexaose			
	Sialic Acid bound HMOs	3′-sialyllactose			
		6′-sialyllactose			
		Disialyllacto-N-hexaose			
		Sialyl-lacto-N-tetraose b			
		Sialyl-lacto-N-tetraose c			
		Disialyllacto-N-tetraose			
	HMOs not bound to Fucose or Sialic Acid	Lacto-N-hexaose			
		Lacto-N-tetrose			
		Lacto-N-neotetraose			
Proteins Targeted	Chemokines, cytokines, growth factors, immunoglobulins, etc	Calprotectin	Electrochemiluminescence (ECLIA), Meso Scale Discovery (MSD) serology kits	(79)	Bode Lab, UC San Diego (USA)
		IgA			
		FSH			
		LH			
		Insulin			
		Leptin			
		FGF-21			
Proteome^b^ Untargeted	Many categories of proteins	Exploratory, over 2,000 proteins identified	LC-MS	(80, 81)	Precision Biomarker Laboratories*(USA)
Metabolites Targeted	Water soluble vitamins	Choline	LC-MS/MS (MxP® Quant 500 kit)	(71, 77, 82)	Biocrates Life Sciences (Austria)
	Alkaloid	Trigonelline			
	Amine Oxide	TMAO (Trimethylamine N-oxide)			
	Free Amino Acids	20 analytes			
	Amino Acid related	30 analytes			
	Bile Acids	14 analytes			
	Biogenic Amines	9 analytes			
	Carboxylic Acids	7 analytes			
	Cresols	p-Cresol-SO4 (p-Cresol sulfate)			
	Free Fatty Acids	12 analytes			
	Hormones & related	4 analytes			
	Indoles & Derivatives	4 analytes			
	Nucleobases & related	Hypoxanthine and Xanthine			
	Carbohydrates & related	Hexoses, including glucose	FIA-MS/MS (MxP® Quant 500 kit)		
	Acylcarnitines	40 analytes			
	Lysophosphatidylcholines	14 analytes			
	Phosphatidylcholines	76 analytes			
	Sphingomyelins	15 analytes			
	Ceramides	28 analytes			
	Dihydroceramides	8 analytes			
	Hexosylceramides	19 analytes			
	Dihexosylceramides	9 analytes			
	Trihexosylceramides	6 analytes			
	Cholesteryl Esters	22 analytes			
	Diglycerides	44 analytes			
	Triglycerides	242 analytes			
Metabolome Untargeted	Many categories of metabolites	Exploratory, 1,000’s of metabolites; known and unknown molecule identities	Rapid LC-MS	(83)	Sapient Bioanalytics (USA)
Microbiota Untargeted	Bacteria	Exploratory, 1,000’s of microbes (~species)	16S rRNA gene sequencing	(84, 85)	Baylor College of Medicine CMMR (USA)

^a^Targeted, analysis of specific known analytes; Untargeted, exploratory analysis of potentially unidentifiable analytes. ^b^Undertaken for a subset only. USDA-ARS WHNRC, United States Department of Agricultural Research Service, Western Human Nutrition Research Center. MILC, Manitoba Interdisciplinary Lactation Center. CMMR, Alkek Center for Metagenomics and Microbiome Research. FGF-21, Fibroblast growth factor-21; RANTS, Regulated on activation, normal T cell expressed and secreted; VEGF-A, Vascular endothelial growth factor-A.

#### 2.6.1 Macronutrients

Total protein, fat and carbohydrates (primarily lactose) will be analyzed by near infrared spectroscopy (NIR) using a SpectraStar XT (KPM analytics, Westborough, MA, United States) calibrated for HM similar to methods previously described (69, 70). This analysis will be performed at the United States Department of Agriculture (USDA) Agricultural Research Service (ARS)-Western Human Nutrition Research Center (WHNRC) in Davis, CA, United States (ELICIT and VITAL-L) or at the MILC biorepository in Canada (CHILD and MISAME). To ensure comparability of results between laboratories, the analysis will be performed in the same manner using the same instrument at each laboratory. A between-lab validation comparison will also been conducted.

#### 2.6.2 Micronutrients

Micronutrients will be analyzed at the USDA ARS-WHNRC using the same methods described for the Mothers, Infants and Lactation Quality (MILQ) study as previously described (71). This includes multiple B-vitamins (72–74), fat-soluble vitamins (vitamin A, vitamin E and carotenoids) (75), and minerals (calcium, copper, iron, magnesium, potassium, selenium, sodium, and zinc) (76) (Table 5). Choline, a water-soluble compound that plays roles in common metabolic pathways with B-vitamins, will be analyzed by liquid chromatography–tandem mass spectrometry (LC–MS/MS, Biocrates Life Sciences, Austria) as part of the MxP® Quant 500 targeted metabolomics analysis described below (77).

#### 2.6.3 Human milk oligosaccharides

Human milk oligosaccharides (HMOs) will be isolated by high-throughput solid-phase extraction, fluorescently labeled, and analyzed by HPLC with fluorescence detection (HPLC-FLD) as previously described (38, 78). Quantification of HMOs is based on retention times and mass spectrometry, with raffinose being used as an internal standard for absolute quantification (molar and mass concentration). This analysis will be done by the Mother-Milk-Infant Center of Research Excellence (MOMI CORE) at the University of California San Diego, United States.

#### 2.6.4 Bioactive proteins—targeted

Specific proteins with known bioactive properties will be quantified by high-performance electrochemiluminescence (79), using kits from Meso Scale Discovery (MSD, United States) at the MOMI CORE. Proteins with similar concentration ranges are multiplexed where possible. Following optimization using HM, three panels have been created for the analysis of: (1) Secretory Immunoglobulin A, (2) Calprotectin, (3) Follicle-Stimulating Hormone (FSH), Luteinizing Hormone (LH), Insulin, Leptin, and Fibroblast growth factor-21 (FGF-21).

#### 2.6.5 Proteome—untargeted

An exploratory proteomics analysis will be performed for a subset of samples at the Precision Biomarker Laboratories (Cedars-Sinai, California, United States) using data independent acquisition mass spectrometry (DIA-MS). All samples will be processed using a S-Trap digestion workflow optimized for HM (80). Samples will then be run on the U3000-Exploris 480 (LC-MS) instrument for quantification of peptides and proteins using DIA-MS method. Each HM sample will be analyzed using a 30-min dual trap optimized workflow (81). Internal indexed retention time standards (iRt’s) will be included in QC and process control pooled samples to monitor system suitability and reproducibility. The acquired proteomic dataset will then be extracted and processed to provide peptide and protein identification.

#### 2.6.6 Metabolites—targeted

LC-MS/MS will be used to analyze 106 metabolites (13 small molecule classes), and flow injection analysis-tandem mass spectrometry (FIA-MS/MS) will be used to analyze 524 metabolites (12 lipid classes and hexoses), using the MxP® Quant 500 kit at biocrates life sciences in Innsbruck, Austria. These methods have been optimized and validated on HM as previously described (71, 77, 82). Briefly, these methods enable absolute quantification (micromolar concentrations) of known metabolites including 242 triglycerides, 12 free fatty acids (not bound to glycerol), 20 free amino acids (not peptide bound), and 40 acylcarnitines. To optimize for HM analysis, samples are thawed on ice and briefly vortexed before measurement; they are not skimmed.

#### 2.6.7 Metabolome—untargeted

An exploratory analysis of metabolites with known and unknown identities will be performed using rapid liquid chromatography-mass spectrometry (rLC-MS) at Sapient Bioanalytics (San Diego, United States) as previously described (83). Briefly, prior to analysis, HM samples are preprocessed by placing on an orbital shaker at 550 rpm at 4°C for 10 min. A 20uL aliquot of sample is transferred to a 96-well microtiter plate containing 80uL of extraction solution, that includes internal standards. Samples are shaken at 550 rpm at 4°C for 10 min followed by centrifugation at 6,000 g at 4°C for 10 min. Supernatant is then transferred to a 384-well polypropylene plate containing 35:65 or 75:25 methanol:water (for positive and negative mode analysis, respectively). Optimization of extraction solvent, sample dilution solution, and final sample concentration for HM was performed using a pooled HM sample.

#### 2.6.8 Microbiota

The microbiome will be analyzed by 16S rRNA gene sequencing of the V4 hypervariable region at the Alkek Center for Metagenomics and Microbiome Research at Baylor College of Medicine (Houston, United States) (84). HM is processed for this analysis with consideration for its low-biomass, and using similar methods compared to those described previously (85), including the use of negative controls (DNA free water) to assess contamination in all processes including initial aliquoting. Prior to library preparation and sequencing, DNA is extracted from the pellet produced by centrifuging 1 mL HM at 10,000 *xg* for 5 min, using the DNeasy 96 PowerSoil Pro HT kit and QIagen QIACube HT automated extraction platform. Data generated from this analysis are exploratory and abundances are relative.

### 2.7 HM data curation

#### 2.7.1 Quality control

Given the complexity and sensitivity of omics data, especially when collected from multiple sites, systematic variations can obscure biological signals. To minimize these technical artifacts, QC approaches are implemented at three levels as described below.

##### 2.7.1.1 QC by analytical laboratories

Each analysis lab has validated their assay for HM (Table 5), and will also perform QC assessments based on the standards of their respective fields. These include systems performance QC [e.g., for rLC-MS, peak mass accuracy during calibration is <5 ppm (83); for near infrared spectroscopy, regular alignments are performed to certified optical standards (70)] as well as analytical QC [e.g., for rLC-MS, isotopically labeled internal standards (83); for microbiome sequencing, use of DNA-free water as a negative control and standard algorithms to identify potential contamination (85)]. Further, all assays will employ technical replicates of a HM pool (section 2.4) to assess whether intra-plate (within-assay) and inter-plate (between-assay) technical variation are below the accepted thresholds for each analytical field.

##### 2.7.1.2 QC by IMiC data analysts

Additional quality assessments of HM data will be performed by IMiC analysts. These include the re-evaluation of technical variation, and evaluations of batch effects by comparison of technical replicates between batches, and where applicable, evaluation of global batch effects using Principal Component analysis (PCA). The type and level of data missingness, data sparsity and distributions are also evaluated and used to inform modality-specific missing value imputation downstream.

##### 2.7.1.3 QC by machine learning pipelines

Further systematic QC is also integrated into the machine learning pipeline developed by the Aghaeepour Lab. This QC will include various strategies to address methodological differences between sites such as in HM sample collection and processing (e.g., collection timepoints, breast washing, method of milk expression, time until freezing). Specifically, unsupervised dimension reduction techniques such as PCA and t-Distributed Stochastic Neighbor Embedding (tSNE) are employed. These methods are adept at revealing clusters within high-dimensional data, allowing for the identification of variation attributed to differences in collection sites. A supervised analysis will also be conducted with the intent to “predict” the collection site of each sample and quantify the magnitude of potential inter-site batch effects. To ensure that final results are reflective of true biological variance rather than methodological discrepancies, statistical analysis pipelines (section 2.8) will implement methods that rigorously control for confounders such as study site. This statistical adjustment will be performed rather than normalization for inter-site differences to minimize modifications to the data that may mask biological effects, considering potential inter-site differences of biological relevance. Additionally, since these statistical approaches may be insufficient to control for site differences in some cases (e.g., for exposures that are completely absent at some sites—such as cesarean delivery, exposures that have fundamentally different meanings across sites—such as birth season, and exposures that have different confounding structures across sites—such as maternal BMI) site-specific analyses are also planned. The evaluation of consistency in within-site associations across the sites, in spite of methodological differences, is also of interest.

#### 2.7.2 Data preprocessing

Data preprocessing will be based on recommendations from analytical labs according to the standard practices of each analytical field, data QC assessments, and the planned data analysis approach. Preprocessing may include: (1) corrections for technical effects, which can include between-plate or batch normalizations, (2) removal of contaminants, (3) removal of analytes considered too sparse (too many zero values) or with too many missing values, and (4) missing data imputation algorithms for left-censored data [e.g. using the limits of detection (LOD)], and for randomly missing data. Details and specific examples are provided below.

##### 2.7.2.1 Batch effects

Generally when batch effects are suspected, data analysts will follow-up with analytical labs to determine next steps. Using targeted metabolomic data as an example, Biocrates analysts use the HM pool included in each plate to perform a median-normalization to the HM pool on a per-analyte basis to account for inter-plate technical variation. When statistical approaches are insufficient, we will consider re-analysis of samples from a specific batch where technical variation is unacceptably high.

##### 2.7.2.2 Decontaminating HM microbiome data

For HM microbiome data, which are prone to contamination issues due to the low biomass of bacteria in HM (86), *Decontam* and *SCruB* algorithms will be used to identify and remove contaminants introduced at both the DNA extraction and sequencing stages (87, 88). These algorithms use information on feature prevalence (88), and batch and position assignment during sequencing to estimate leakage of sequences across samples (87).

##### 2.7.2.3 Missing data—filtering and imputation

Missing data issues are prevalent in omics data, stemming from various factors such as cost, poor sample quality, inadequate sample volume, instrument or assay detection limitations, or other experimental factors. Based on the mechanisms producing missing values, these unrecorded data can be further classified as missing not at random (MNAR) or missing at random (MAR) (89). Missing data can be imputed, or the entire feature can be removed.

To avoid introducing large biases when minimal data are available for a given analyte/feature, we will implement preliminary feature- or sample-filtering for specific data types based on standard practices of analytical fields, with consideration for planned downstream analyses. Generally across all study sites and modalities, if over 30% of a feature’s values are considered MAR within any time-point and arm, the feature will be removed.

For targeted metabolomic data, rather than completely removing features with few non-missing values, features with more than 20% of values below the LOD in any given time-point and intervention arm will be binarized across all sites, such that they are retained in the machine learning analysis as it can handle binary features. For untargeted metabolomic data, only metabolites with high alignment confidence between batches (based on mass to charge ratios) will be used in the integrated analysis.

For HM microbiome data, we previously found a depth of 8,000 reads to be sufficient to capture the diversity of HM microbiota (27). Samples with fewer reads are typically removed from analysis. For the current study, this threshold will be relaxed to 1,000 reads to maximize sample retention for integrated analysis. However, to account for the expected lower accuracy of community composition estimates for samples with low read count, an indicator variable identifying samples with fewer than 8,000 reads will be used for adjustment. Further, only microbial features present in over 10% of samples in each site and at each time-point will be included.

After the sample- and feature-filtering described above, any remaining missing values will be imputed. To impute left- and right-censored MNAR data that are missing due to being below or above the limit of detection (LOD) of a given assay, respectively, we will generally follow recommendations from the responsible analytical lab. For microbiome data, zero values (considered left-censored MNAR) will be imputed using Bayesian-multiplicative replacement (90). For untargeted metabolomics data and targeted protein data, left-censored missing values will be imputed by following a uniform distribution with the lower bound set to one-tenth of the minimum observed value and the upper bound to the minimum observed value. Targeted protein values above the detection range (right-censored) will be imputed using a log normal distribution. For targeted metabolomics data, values below the LOD will be imputed using logspline density estimation (91), and using consensus LOD values across plates within each batch. Further, we will interpolate values considered to be MAR with a non-parametric multivariate model based on random forests using the *MissForest* package (92). At each iteration, every feature with existing blank entries will be taken as the outcome predicted by other features. This methodology allows for the elucidation of nonlinear relationships between features, leveraging the interconnected dependencies inherent in biomolecular entities.

##### 2.7.2.4 Data transformations

Data transformations will generally not be performed given the use of a late fusion model, where each individual model is designed to handle different data distributions independently. Exceptions include the binarization of some features with few non-missing values as described above, and a centered-log ratio (CLR) transformation of microbiome features, used to account for the compositionality issue of sequencing data (93).

### 2.8 Data management

#### 2.8.1 Initial data contribution and management

**Figure 3:**
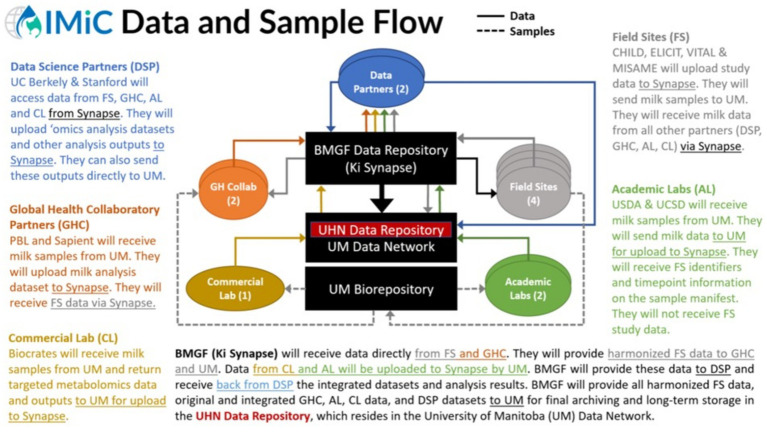
Data and sample flow for the International Milk Composition (IMiC) Consortium. AL, Academic Labs; BMGF, Bill & Melinda Gates Foundation; CL, Commercial Labs; DSP, Data Science Partners; FS, Field Sites; GHC, Global Health Collaboratory; Ki, Knowledge Integration; UHN, University Health Network; UM, University of Manitoba.

Data contribution and access for IMiC are illustrated in Figure 3 and governed by data sharing agreements. All PII is removed from the data prior to contribution. Briefly, each study provides its required data for IMiC harmonization (a subset of their entire study dataset) to the Ki team at the Bill & Melinda Gates Foundation via secure upload to their Synapse platform. Similarly, each HM analysis lab securely uploads their data to Synapse, or securely transfers their data to the University of Manitoba for upload to Synapse. Synapse is a secure collaborative compute space that allows scientists to share and analyze data together.^2^ Data housed within the Ki Synapse platform are not available or open to the public.

#### 2.8.2 Long-term data management

For long-term data management and storage, a bespoke IMiC Database will be developed by the University Health Network and housed within the University of Manitoba’s Secure Research Environment. All final IMiC datasets will be transferred from Synapse to the IMiC Database. Standardized file descriptions and data dictionaries will be used across studies. A user-friendly browser-based interface will enhance accessibility for authorized users. This database will support long-term management and utilization of IMiC data, and can be expanded to integrate similar datasets from future HM studies, offering opportunities for cross-comparison and meta-analysis.

#### 2.8.3 Data access and availability

Initially, data access will be managed on the Ki Synapse platform and restricted to IMiC members according to their assigned role. For example, core data analysts require access to all datasets from all studies, while individual study investigators require access only to their own study data and HM data. Following publication of primary IMiC results, the final IMiC dataset will be available for secondary analysis upon request via the IMiC database, in alignment with the parameters of original informed consent of each study.

### 2.9 Data analysis

#### 2.9.1 Primary exposures and outcomes

The main exposure and outcome variables of interest to IMiC can be structured into categories based on the research questions summarized in Box 1. Our two overarching hypotheses are: (1) maternal and environmental factors (exposures) affect HM composition (outcome), and (2) HM composition (exposure) affects infant growth (outcome). These hypotheses can be explored independently, but we also aim to integrate them, and estimate how various states of maternal nutrition affect the predicted impact of milk composition on infant growth. Overall, maternal nutritional status is an exposure with indicators including maternal anthropometrics (BMI, MUAC) and nutritional interventions. Other exposures for hypothesis 1 include maternal age, parity and indicators of the physical and social environment. HM composition is the outcome for hypothesis 1, but is also the primary exposure for hypothesis 2. Meanwhile, infant growth is the primary outcome of interest, and the main indicators of this will be length for age z-scores (LAZ), weight for age z-scores (WLZ), and weight for length z-scores (WLZ). Given the importance of wasting (WLZ < −2) and stunting (LAZ < −2) as indicators of chronic undernutrition, the prevalence of these conditions at 3, 6, 12, and 18 months of age will also be predicted from integrated-omic models to explore if associations with milk composition differ compared to continuous growth measures.

#### 2.9.2 Integrated analysis of HM composition and infant growth

We aim to use an integrative multiomics approach to increase the accuracy of a model predicting infant growth outcomes from HM components, and to identify novel molecular pathways associated with the predicted growth outcomes. A machine learning pipeline developed by the Aghaeepour Lab will be used to address this aim and assess whether the integrative multiomics approach increases model accuracy for predicting growth outcomes. A more complete analysis plan is described in the supplement (Supplementary material). Briefly, we will predict infant growth outcomes and baseline risk factors while controlling for potential confounders using algorithms tailored to each modality that implement eXtreme Gradient Boosting (XGBoost) (94, 95). Models will be validated using a customized two-layered cross-validation strategy designed to ensure the models’ robustness and generalizability, assessed using performance metrics such as AUROC, AUPRC, or Spearman’s *ρ*. Key milk components of interest will be identified using a feature selection process that combines forward selection and backward elimination. Further, a late fusion technique will be used to integrate omics modalities and epidemiological data in a combined model. Lastly, pathway enrichment analysis will be conducted to aid in the biological interpretation of predictive models, and to potentially identify molecular mechanisms that underlay the effect of HM components on infant growth.

#### 2.9.3 Intervention effects analysis

We will also estimate the effects of randomized interventions on human milk components in the three intervention studies. Two different definitions of ‘milk composition’ will be used (1) individual milk components, adjusted for multiple testing and (2) predictive summary measures calculated from milk biomarkers of infant growth using targeted learning and other dimension reduction techniques (96). We will estimate both unadjusted and covariate-adjusted intervention effects on each HM outcome, adjusting for baseline prognostic factors using cross-validated targeted maximum likelihood estimation (97). In addition to analyzing HM components at each time point, we will also examine changes in HM components between timepoints. We will correct for multiple comparisons by using the Benjamini-Hochberg procedure, and group corrections by time-point and outcome group. Primary outcomes are macro- and micro-nutrients, secondary outcomes are HMOs and targeted proteins and bioactives, tertiary outcomes are targeted metabolomics, and exploratory outcomes and untargeted metabolomics, untargeted proteomics, and the microbiome.

As a secondary analysis, we plan to assess the potential mediating effects of HM components on the relationship between maternal nutritional supplementation or other maternal factors, and infant growth outcomes (Supplementary material).

### 2.10 Consortium operations

#### 2.10.1 Guiding principles of collaboration

Data sharing, subawards, and intellectual property ownership are governed by legal agreements between the University of Manitoba, the Bill & Melinda Gates Foundation and other participating institutions. The overall guiding principles of IMiC are outlined in the IMiC Consortium Agreement, a non-legally-binding document that was co-developed by consortium members in order to promote collaboration, transparency and equity among all members. The Consortium Agreement describes the overall goals and vision of IMiC, its governance structure (including an external scientific advisory committee), and its general principles of collaboration, confidentiality, equity, expediency, data stewardship and research integrity. This agreement also outlines policies and expectations related to data sharing and confidentiality, publication and authorship, communications and intellectual property ownership.

#### 2.10.2 Participant and public involvement

As a consortium undertaking a secondary analysis of four separate studies, IMiC will not directly engage participants or the public in study design, conduct and reporting. However, each study followed their own protocols locally regarding engagement, and study representatives were encouraged to bring these perspectives to IMiC discussions. Moreover, we plan to communicate IMiC research results to the public and participants through each study’s local team.

## 3 Discussion

Taking a collaborative, multi-disciplinary and multi-omic approach to HM science, IMiC will provide new insights on the sources and consequences of the tremendous variation in human milk composition across populations. These findings will fill gaps (7–9) and advance knowledge about how HM operates as a biological system to support infant development, and identify known and novel HM components and “profiles” that could be leveraged to develop new approaches to optimizing infant nutrition and growth in diverse settings.

### 3.1 Analytical innovations

While there is an increasing appreciation for the need to study HM as a biological system (6, 44), this concept is still relatively new and few studies have taken this approach. The IMiC team is rising to the challenge and promises to deliver innovative approaches and novel findings. By measuring and integrating a broad collection of HM components, and applying advanced machine learning techniques, we will have unprecedented opportunities to infer and investigate biological pathways and mechanisms. By including exploratory assays, we have the potential to discover new HM components relevant to infant growth. By analyzing HM from maternal nutritional intervention studies, we can determine if HM mediates the impact of these interventions on infant growth—if so, we can pinpoint which component(s) are responsible.

### 3.2 Challenges

General challenges for the IMiC consortium include: (1) the administrative burden and complexity of financial and legal agreements for subgrants, data sharing and/or material transfer across 13 institutions; (2) the logistical burden and complexity of shipping frozen human milk across countries and continents; (3) communication challenges related to geographic and cultural diversity; (4) data harmonization challenges stemming from the different study designs and data collection time points; and (5) ensuring equitable representation, participation, collaboration and attribution for all members, including those from low-middle-resource and low-middle-income country (LMIC) settings. The latter has been the primary focus of the IMiC LMIC working group, whose work will be described elsewhere.

Selection bias and generalizability are challenges of particular note in our study, where participants were selected partially based on data availability across independent studies across diverse settings. For instance, only participants able to provide milk samples and attend follow-up visits for infant anthropometric measurements could be included, and unmeasured maternal or socioeconomic factors could differ between those able to participate and all eligible participants, leading to potential biases in the associations identified (98). We will implement strategies to mitigate selection bias during the data analysis phase (e.g., through careful consideration of handling missing data) and will consider its unintended effects during the knowledge translation phase, such as lack of transferability of some conclusions across geographic settings. We will also be sure to use the most relevant reference standards for the diverse populations we are studying (e.g., using mid upper arm circumference rather than BMI to identify maternal undernutrition, and using international infant growth standards that have been validated across different ethnic groups).

Additionally, the overall IMiC project was delayed by approximately 18 months due to the COVID-19 pandemic. Two studies (MISAME and VITAL) were also delayed in completing their recruitment and data collection, several analytical laboratories were shut down, and supply-chain disruptions affected the availability of materials and reagents. The individual studies participating in IMiC each faced distinct challenges that are beyond the scope of this paper (e.g., recruiting and standardizing protocols across multiple towns or provinces; logistical challenges in low-middle-resource settings related to collecting samples, maintaining cold chain, receiving shipments and resolving taxation issues).

### 3.3 Future opportunities

Beyond the analytical plans described here, there are additional research questions that could be addressed with IMiC data. For instance, there is an opportunity to assess maternal and environmental determinants of “response” to nutritional supplementation (i.e., identify mothers whose HM composition changes more or less with treatment). Further, beyond the data described here, additional clinical and biological data are available from the IMiC studies, offering opportunities to build on initial IMiC results to pose further questions about the relationships between HM and infant development. For example, it will be possible to investigate how different HM profiles or components relate to longer-term growth outcomes (up to 13 years in CHILD), stool microbiome composition (CHILD, MISAME, VITAL, ELICIT), enteric pathogen colonization (ELICIT, VITAL), urine metabolomics (ELICIT, CHILD) and maternal genetics (CHILD). Notably, the link between milk composition and maternal genetics can only be explored within the CHILD Cohort study and ELICIT trial, since consent for genetic analysis was not included in the original study protocols for the other studies.

Further, beyond the 4 currently participating populations, IMiC has the potential to serve as a platform for HM research in other populations, addressing innumerable research questions. By assembling a multidisciplinary network of research teams dedicated to HM science, developing quality control reagents, and validating new assays for HM analysis, IMiC has established a comprehensive pipeline that can be flexibly applied to study HM as a biological system in any context. For example, initiatives are already underway to leverage the IMiC infrastructure to study how HM shapes microbiome and immune development in healthy Canadian children, how HM can optimally support very low birth weight and premature infants, and how HM drives neurodevelopment in the context of socioeconomic deprivation.

### 3.4 Strengths and limitations

Key strengths of the study include the large sample size (1,040 dyads, 1,946 HM samples), the diverse study population (4 countries, including 3 low-middle resource countries), and the comprehensive, harmonized and specialized approach to HM analysis, with all samples analyzed on the same platforms using HM-validated assays for a multitude of nutritive and non-nutritive components using both targeted and untargeted approaches. To our knowledge, no prior study has performed such a comprehensive analysis of HM on this scale and several of the IMiC assays have not previously been validated for HM. The retroactive harmonization of independent studies poses a data harmonization challenge, but also provides robust opportunities for validation of novel discoveries through a meta-analytic approach. Other limitations relate to the HM collection protocols, which varied across studies in terms of methods and time points, impacting our ability to meaningfully compare certain components across studies. Finally, HM volumes produced by mothers and/or consumed by infants were not captured, which prevents us from quantifying the amount (“dose”) of each HM component ingested.

### 3.5 Conclusion

Through its innovative and multidisciplinary approach to studying HM as a biological system, the IMiC consortium will advance our understanding of HM composition, its variation across settings, and the sources and consequences of this variation for infant growth. Further, it will serve as a template for future HM research, offering rich opportunities for collaboration, training and discovery across disciplines and global settings.

## 4 Ethics and dissemination

### 4.1 Ethics

The IMiC project, involving the secondary analysis of HM collected by the four participating studies, was reviewed and approved by the human Health Research Ethics Board (HREB) at the University of Manitoba on April 20, 2020 (Approval ID: HS23767). Each participating study, including protocols for the collection of HM and metadata, received ethical review and approval at their primary institution(s). Voluntary informed consent was obtained from all participants before or at enrollment. Informed consent procedures are described in individual study protocols (48–51, 57). Briefly, participants provided written signed consent in the local language. Alternatively, in cases of illiteracy in MISAME-3 and VITAL-LW, a thumb impression was asked of the participant and witnessed. For eligible participants, the details of the specific trial, including study procedures, were explained by team members or project midwives regardless of literacy. The CHILD study was approved by the University of Alberta, University of British Columbia, University of Manitoba and McMaster University Human Research Ethics Boards. The ELICIT Study was approved by Tanzania’s National Institute of Medical Research, the University of Virginia Health Sciences Research Institutional Review Board, and the Tanzanian Food and Drug Administration. The VITAL-LW study was approved by the Institution Review Board of VITAL Pakistan Trust, Ethics Review Committee of Aga Khan University, and National Bioethics Committee of Pakistan. The MISAME-3 Study was approved by the University Hospital of Ghent University and the Burkinabe ethics committee.

### 4.2 Knowledge translation

Results from IMiC will be disseminated through traditional academic platforms (e.g., presentation at scientific conferences and Open Access publication in peer-reviewed journals) and promoted through members’ academic, clinical and social networks. IMiC members and advisors include academic and clinician researchers with contacts and/or volunteer positions in relevant societies such as the International Society for Human Milk and Lactation and relevant guideline developing organizations such as the World Health Organization. IMiC is also well positioned to translate new discoveries into testable hypotheses for mechanistic research, and candidate products to support maternal and infant nutrition. Finally, research results will be shared with participants through each study’s local team in accordance with their individual policies and practices (e.g., participant-directed newsletters, websites, social media).

## Footnotes

^1^https://www.kiglobalhealth.org/.

^2^ https://www.synapse.org/#
