# Supplementary material for: Protocol: the International Milk Composition (IMiC) Consortium - a harmonized secondary analysis of human milk from four studies
Source: Front Nutr. 2025 Jun 10;12:1548739. doi: 10.3389/fnut.2025.1548739 (PMC12186657; doi:10.3389/fnut.2025.1548739)
Supplement: Supplementary file 1 [file Data_Sheet_1.pdf]

Supplementary Material

1 List of Supplementary Material

Table S1. Harmonization spec sheet showing available variables in the international milk composition consortium (IMiC) study.

Figure S1. Distribution of human milk samples and quality control (QC) milk pool replicates from study field sites to laboratory analysis sites.

Supplemental File 1. Standard operating protocol for aliquoting milk samples and generating milk pools in the IMiC study.

Supplementary Note 1. Multi-omics integrated machine learning analysis to assess the effect of HM composition on infant growth outcomes.

Supplemental Note 2. Targeted learning mediation of infant growth outcomes through human milk composition and effect modification

2 Supplementary Figures

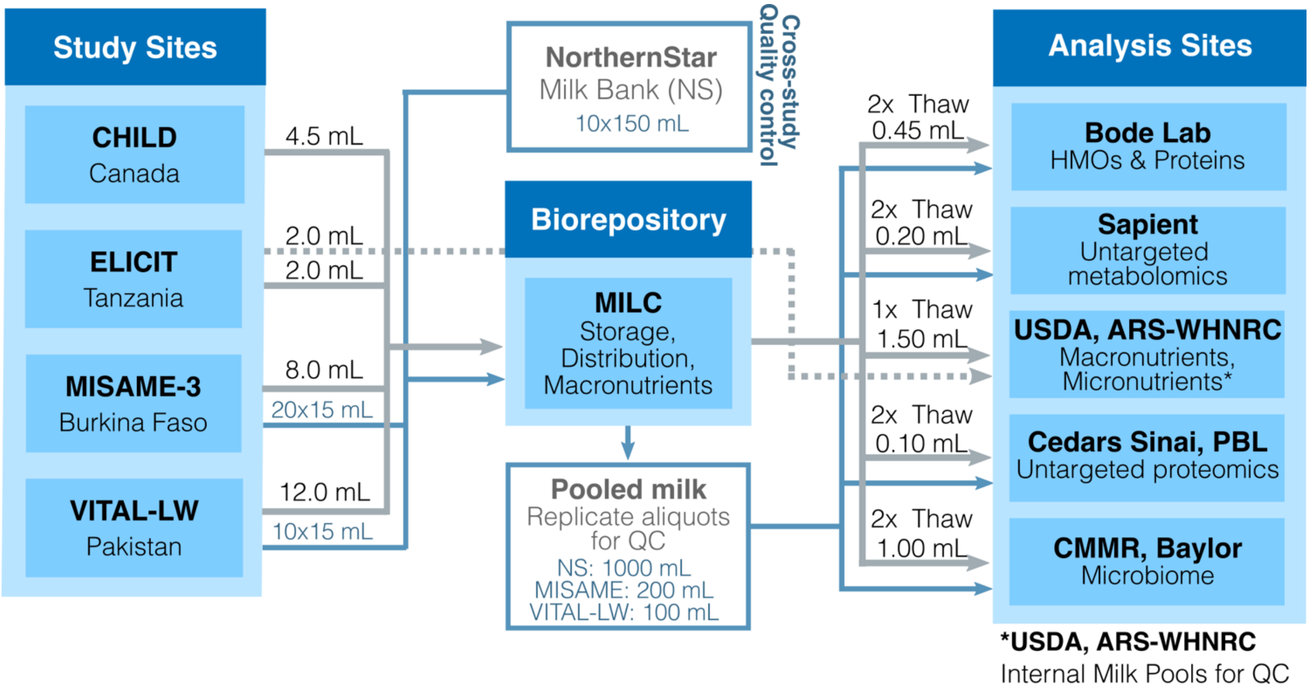

Figure S1. Distribution of human milk samples and quality control (QC) milk pool replicates from study field sites to laboratory analysis sites. Gray lines indicate paths of samples (volumes are per-sample) and blue lines indicate paths of milk for quality control. Note that an ELICIT aliquot went directly to the USDA, ARS-WHNRC (dashed line) rather than going through the MILC biorepository first. Macronutrient analysis of CHILD and MISAME-3 samples was done at the MILC Biorepository after sub-aliquoting (2x Freeze-Thaws), and macronutrient analysis of ELICIT and VITAL-LW samples were done at the USDA, ARS-WHNRC (2x Freeze-Thaws).

3 Supplementary Note 1. Multi-omics integrated machine learning analysis to assess the effect of HM composition on infant growth outcomes.

3.1 Prediction of outcomes and baseline risk factors

We will employ advanced machine learning techniques to predict infant growth outcomes. Specifically, models based on eXtreme Gradient Boosting (XGBoost)(1), a powerful and efficient implementation of gradient boosted decision trees, will be tailored for each omics dataset. The predictive power of XGBoost lies in its ability to handle a variety of data types, deal with missing values, and capture complex nonlinear relationships between features and outcomes. A multi-modal pipeline utilizing late fusion techniques will be constructed for each outcome, where independent models for each omics dataset will be trained towards predicting outcomes separately and integrated to generate a final result(2). This pipeline can not only gauge the predictive power of each dataset individually but also mitigate dimensionality challenges found in other fusion techniques, serving as an ensemble system to leverage the strengths of all omics data within the maternal milk profile.

The performance of these predictive models will be rigorously evaluated using the following statistical measures: the Area Under the Receiver Operating Characteristic (AUROC) and the Area Under the Precision-Recall Curve (AUPRC) for the categorical outcomes and Spearman's ρ for the continuous outcomes. The AUROC is a widely used metric that measures the ability of a model to distinguish between classes—higher values indicate better discrimination. However, in the context of imbalanced datasets, where the outcomes of interest, such as severe wasting or stunting are relatively rare, AUPRC could provide a more relevant information for rare outcomes since it summarizes the trade-off between precision and recall for different thresholds, the AUPRC is more sensitive to the model's performance on the minority class. On the other hand, Spearman's ρ is chosen to assess monotonic associations between model prediction and ground truth due to its robustness to outliers. Together, these metrics will guide the development of the most accurate and clinically useful predictive models.

We will also implement a multitask learning framework to account for the interconnected nature of infant growth outcomes. This methodological approach acknowledges that these conditions may share common underlying biological pathways and risk factors. Multitask learning not only improves the efficiency of the learning process but also has the potential to reveal insights into the shared etiology of these growth outcomes. Empirical evidence from our previous studies suggests that multitask learning can lead to models that outperform those trained on individual tasks, especially in domains like perinatal health, where the interdependence between different conditions is significant (3).

Multisite analysis is a critical component of model building, ensuring that the predictive models are robust and applicable across different populations and conditions. Initial models will be tailored and validated for each site separately to account for variations in demographic, environmental, and procedural factors between different collection sites. This site-specific modeling approach allows for the detection of local patterns and site-specific risk profiles. Once established, these models will then be cross-applied to other sites to test their predictive validity in different settings.

When models developed for a specific site do not generalize well to others—a scenario that might arise due to substantial site-specific differences—the consortium will pivot to constructing shared models that pool data from all sites. This approach leverages commonalities in the data while accounting for site-specific variation through the application of double machine learning technique (4). Double machine learning allows for more accurate causal inference by isolating the effect of interest while controlling for covariates. Such shared models benefit from a larger dataset, improving the statistical power and the potential for capturing a more comprehensive range of biological variations associated with the outcomes of interest.

3.2 Model validation

Model validation will be executed using a customized two-layered cross-validation strategy designed to ensure the models' robustness and generalizability. This rigorous approach to validation is essential to confirm that the models will perform well in real-world clinical settings, where they will be applied to predict infant growth outcomes.

In the inner layer of cross-validation, the objective is to fine-tune the model by optimizing its free parameters. By iterating through different parameter values and evaluating performance, the inner layer selects the parameter set that yields the best results, measured by cross-validated performance metrics such as AUROC, AUPRC, or Spearman's ρ.

The outer layer implements a modified “leave-one-out” cross-validation procedure. Leave-one-out cross-validation involves training the model on all but one sample and then testing the model on that single left-out sample. This strategy ensures that the model is tested on a completely unseen patient's data, thus mimicking the real-world scenario where predictions would be made on new patients not included in the model training. This process is repeated until each patient has been left out once, and the model has been trained and validated on all other data. Such an approach safeguards against overfitting and ensures that the reported performance metrics reflect the model's ability to generalize to new subjects, providing a robust estimate of how the model will perform in clinical practice.

3.3 Feature selection

To identify key milk components of interest, we will implement a feature selection process that combines forward selection and backward elimination. Beginning with forward selection, the models will incrementally add features, assessing their contribution to the model's predictive power. This approach helps to construct a model that optimizes the variance-bias trade-off, and will thus include only the most informative variables, avoiding complexity that does not improve model fit. The inclusion of new features continues until there is no further improvement in the model's performance, indicating that the optimal subset of features has been reached. Following the identification of a provisional feature set, backward elimination is applied to prune the model further. This method systematically removes the least informative features while monitoring the impact on predictive performance. The elimination of features continues until it is determined that any further removal would detrimentally affect the model's accuracy. Through this iterative process, we ensure the development of streamlined models, which, given the inherent limitations of the data (sample size, noise), will be composed of the features most robustly predictive of infant growth outcomes. If this process does not yield a universal set of features across all sites, the consortium will conduct site-specific feature selection to account for local differences, ensuring that the models maintain their precision and relevance in varied contexts.

3.4 Integration with phenotypic data

We plan to integrate biological data with phenotypic metadata to enhance the understanding and prediction of infant growth outcomes. The first method involves utilizing the rich phenotypic data available to construct models that analyze the impact of external factors, such as environmental exposures and lifestyle choices, on biological processes. This will be achieved by correlating omics data with various metadata variables. This approach aims to uncover how external factors influence maternal milk profiles, thereby identifying potential risk factors and elucidating the biological mechanisms through which they operate.

In addition, we will employ the late fusion technique to build combined models that integrate epidemiological data with omics data. The goal is to enhance the predictive power for infant growth outcomes by harnessing the complementary strengths of both data types, leading to more accurate and comprehensive predictive models.

3.5 Pathway enrichment analysis

Pathway enrichment analysis is an essential step for interpreting the results of predictive models in biological terms, particularly when the aim is to understand the molecular underpinnings of infant growth outcomes. For each site and growth outcome, pathway enrichment will be conducted separately

to ensure that the localized biological processes are accurately captured. Furthermore, distinct tools will be implemented for available omics data. For instance, using MetaboAnalyst, a comprehensive tool for metabolomic data analysis and interpretation, the consortium will leverage its rich repository of public curated datasets to map significant features from the predictive models to known metabolic or signaling pathways (5). Top proteomic features will be analyzed utilizing gene set overrepresentation analysis of GO terms (6) with the topGO package in R (7).

4 Supplemental Note 2. Targeted learning mediation of infant growth outcomes through human milk composition and effect modification

The UC Berkeley team will test for mediating effects of HM in relation to interventions and maternal characteristics. Because of the large number of HM components compared to observations, this analysis will only utilize the macro- and micronutrients as well as summary measures of other HM components (i.e. principal components, or microbiome diversity).

Stochastic interventions will be used to estimate how a theoretical intervention would affect the predicted impact of modifying continuous HM components on infant growth. We will do so both in unadjusted fashion and adjust for time-dependent covariates using the Targeted Learning framework (8). The impact of HM composition will be estimated separately for each cohort and a meta-analytic approach will be used to combine estimates across studies to transparently address inter-study heterogeneity. This includes the heterogeneity in the timing and measurement of outcomes (indicators of infant growth) and timing of the exposures (milk composition measurements) among studies.

Directed acyclic graphs (DAGs) will be used to identify potential confounders from the covariates shown in **Table S1** in addition to derived summary variables (9). Missing covariate observations will be imputed as the median (continuous variables) or mode (categorical variables) among all children within each cohort, and an indicator variable for missingness will be included in the adjustment set. Targeted maximum likelihood estimation will be used to estimate the marginal effects of potential interventions adjusting for potential confounders. This method requires estimating both the outcome model and intervention model, and model misspecification is reduced by using ensemble machine learning for both components. This is a two-stage strategy that incorporates both valid statistical inference and a super learner of novel machine learning algorithms (10). The super learner is an ensemble machine learning method that uses cross-validation to select a weighted combination of predictions from a library of algorithms.

Of note, to account for dependence due to clustering in the data, clustered standard errors will be estimated to account for repeated measures when relevant. Further, 95% confidence intervals for incidence will be estimated using the normal approximation. Lastly, for analyses that require estimating many equivalent parameters, such as the associations of many biomarkers and growth outcomes, multiple testing will be adjusted based on the false discovery rate for sets of related associations (11).

5 References

1. Chen T, Guestrin C. XGBoost: A Scalable Tree Boosting System. *Proceedings of the 22nd ACM SIGKDD International Conference on Knowledge Discovery and Data Mining*. New York, NY, USA: ACM (2016) doi: 10.1145/2939672.2939785

2. Athaya T, Ripan RC, Li X, Hu H. Multimodal deep learning approaches for single-cell multi-omics data integration. *Brief Bioinform* (2023) 24: doi: 10.1093/bib/bbad313

3. De Francesco D, Reiss JD, Roger J, Tang AS, Chang AL, Becker M, Phongpreecha T, Espinosa C, Morin S, Berson E, et al. Data-driven longitudinal characterization of neonatal health and morbidity. *Sci Transl Med* (2023) 15:eadc9854.

4. Chernozhukov V, Chetverikov D, Demirer M, Duflo E, Hansen C, Newey W, Robins J. Double/debiased machine learning for treatment and structural parameters. *Econom J* (2018) 21:C1–C68.

5. Chong J, Xia J. MetaboAnalystR: an R package for flexible and reproducible analysis of metabolomics data. *Bioinformatics* (2018) 34:4313–4314.

6. Ashburner M, Ball CA, Blake JA, Botstein D, Butler H, Cherry JM, Davis AP, Dolinski K, Dwight SS, Eppig JT, et al. Gene ontology: tool for the unification of biology. The Gene Ontology Consortium. *Nat Genet* (2000) 25:25–29.

7. Alexa A, Rahnenführer J. Gene set enrichment analysis with topGO. R Package version 2.24.0. (2016) https://bioconductor.org/packages/release/bioc/vignettes/topGO/inst/doc/topGO.pdf

8. Coyle, Hejazi, Malenica, Phillips, Arnold, Mertens, Benjamin-Chung, Cai, Dayal, Colford, et al. Targeting learning: robust statistics for reproducible research. *arXiv preprint arXiv:2006 07333*

9. Greenland S, Pearl J, Robins JM. Confounding and Collapsibility in Causal Inference. *SSO Schweiz Monatsschr Zahnheilkd* (1999) 14:29–46.

10. van der Laan MJ, Polley EC, Hubbard AE. Super learner. *Stat Appl Genet Mol Biol* (2007) 6:Article25.

11. Benjamini, Hochberg. Controlling the false discovery rate: a practical and powerful approach to multiple testing. *J R Stat Soc Series B Stat Methodol* (1995) 57:289–300.
